# Single-cell transcriptomics identifies potential cells of origin of MYC rhabdoid tumors

**DOI:** 10.1038/s41467-022-29152-4

**Published:** 2022-03-22

**Authors:** Monika Graf, Marta Interlandi, Natalia Moreno, Dörthe Holdhof, Carolin Göbel, Viktoria Melcher, Julius Mertins, Thomas K. Albert, Dennis Kastrati, Amelie Alfert, Till Holsten, Flavia de Faria, Michael Meisterernst, Claudia Rossig, Monika Warmuth-Metz, Johannes Nowak, Gerd Meyer zu Hörste, Chloe Mayère, Serge Nef, Pascal Johann, Michael C. Frühwald, Martin Dugas, Ulrich Schüller, Kornelius Kerl

**Affiliations:** 1 Department of Pediatric Hematology and Oncology, University Children’s Hospital Münster, 48149 Münster, Germany; 2 Institute of Medical Informatics, University of Münster, 48149 Münster, Germany; 3 Department of Pediatric Hematology and Oncology, University Medical Center Hamburg-Eppendorf, 20251 Hamburg, Germany; 4 Institute of Neuropathology, University Medical Center Hamburg-Eppendorf, 20251 Hamburg, Germany; 5 Department of Neurology, Schlosspark-Klinik, 14059 Berlin, Germany; 6 Institute of Molecular Tumor Biology, University of Münster, 48149 Münster, Germany; 7 Department of Pediatric Hematology and Oncology, Children’s Hospital of Brasìlia, 70684-831 Brasìlia, Brazil; 8 Neuroradiological Reference Center, University Hospital Würzburg, Würzburg, Germany; 9 SRH Poliklinik Gera GmbH, Radiological Practice Gotha, Gotha, Germany; 10 Department of Neurology with Institute of Translational Neurology, University Hospital Münster, 48149 Münster, Germany; 11 Department of Genetic Medicine and Development, University of Geneva, 1211 Geneva, Switzerland; 12 iGE3, Institute of Genetics and Genomics of Geneva, University of Geneva, 1211 Geneva, Switzerland; 13 Swabian Children’s Cancer Center, Paediatric and Adolescent Medicine, University Medical Center Augsburg, 86156 Augsburg, Germany; 14 Division of Pediatric Neurooncology, German Cancer Consortium (DKTK), German Cancer Research Center (DKFZ), Heidelberg, Germany; 15 Institute of Medical Informatics, Heidelberg University Hospital, Heidelberg, Germany; 16 Research Institute Children’s Cancer Center, 20251 Hamburg, Germany

**Keywords:** Targeted therapies, Embryonal neoplasms, Paediatric cancer, Tumour heterogeneity, Transcriptomics

## Abstract

Rhabdoid tumors (RT) are rare and highly aggressive pediatric neoplasms. Their epigenetically-driven intertumoral heterogeneity is well described; however, the cellular origin of RT remains an enigma. Here, we establish and characterize different genetically engineered mouse models driven under the control of distinct promoters and being active in early progenitor cell types with diverse embryonic onsets. From all models only *Sox2*-positive progenitor cells give rise to murine RT. Using single-cell analyses, we identify distinct cells of origin for the SHH and MYC subgroups of RT, rooting in early stages of embryogenesis. Intra- and extracranial MYC tumors harbor common genetic programs and potentially originate from fetal primordial germ cells (PGCs). Using PGC specific Smarcb1 knockout mouse models we validate that MYC RT originate from these progenitor cells. We uncover an epigenetic imbalance in MYC tumors compared to PGCs being sustained by epigenetically-driven subpopulations. Importantly, treatments with the DNA demethylating agent decitabine successfully impair tumor growth in vitro and in vivo. In summary, our work sheds light on the origin of RT and supports the clinical relevance of DNA methyltransferase inhibitors against this disease.

## Abstract (web summary)

Rhabdoid tumors (RT) are aggressive paediatric cancers with yet unknown cells of origin. Here, the authors establish genetically engineered mouse models of RT and, using single-cell RNA-seq and epigenomics, identify potential cells of origin for the SHH and MYC subtypes.

## Introduction

Rhabdoid tumors (RT) are rare and aggressive embryonic tumors of infancy and early childhood associated with a poor outcome, especially after relapse^1–3^. RT arise in different anatomic locations, most frequently in the central nervous system (CNS) (Atypical Teratoid Rhabdoid Tumor, ATRT), the kidney (Rhabdoid Tumor of the Kidney, RTK) or soft tissues (Malignant Rhabdoid Tumor, MRT)^4–6^. Biallelic mutations in the gene *SMARCB1* (>95% of cases), or rarely *SMARCA4* (<5%), are sufficient to provoke tumor formation. Beyond that, RT show remarkable genetic homogeneity^7,8^. Both genes encode core subunits of the SWI/SNF chromatin remodeling complex, which regulates cellular processes such as proliferation, differentiation, and stem cell maintenance^9,10^. Hence, these mutations cause genome-wide downstream effects foremost in the epigenome and ultimately the transcriptome and proteome^11^.

While showing a low mutational burden, RT are characterized by a molecular heterogeneity which includes 5 stable methylation subgroups: the three pre-known ATRT-SHH (activated sonic hedgehog signaling), ATRT-MYC (*MYC* oncogene-driven), and ATRT-TYR (active melanoma-associated genes)^7^, as well as two novel groups, group 3 and group 4^11,12^. The latter comprise extracranial cases resembling the ATRT-MYC phenotype; however, they differ in their localization, differential pathway enrichment, and global DNA methylation. Re-analysis of existing subgrouping approaches led to a unified consensus classification^12,13^.

RT include different morphological phenotypes even within the same tumor, e.g., undifferentiated tumor cells with occasional areas of primitive neuroepithelial-like, epithelial or mesenchymal differentiation^4,6,14^. So far, distinct precursors have been suggested for the three RT subgroups. For instance, ATRT-SHH are proposed to derive from neural progenitors, while RT of the MYC subgroup and ATRT-TYR having a more mesenchymal phenotype, are suggested to derive from neural crest cells and mid/hindbrain progenitors^15–19^.

The molecular diversity and vulnerable time frame of tumor manifestation during mouse embryonic development^16,18,20^ suggest that RT either arise from multipotent progenitor cells or are prone to reprogramming or transdifferentiation. A highly dynamic cell pool present during the vulnerable period of RT induction are primordial germ cells (PGCs), which are multipotent, epigenetically regulated and give rise to the gametes upon differentiation^21,22^. It has been widely accepted that intracranial germinomas originate from aberrantly migrated, transdifferentiated PGCs during development^23,24^.

Here, we used genetically engineered mouse models (GEMMs) and single-cell RNA sequencing (scRNA-seq) technology to unravel the cellular origin of RT. We show that *Sox2*-positive progenitor cells give rise to murine RT of the MYC and SHH subgroups. Using similarity approaches with reference cell types of early to mid-stages of the embryo, we found that murine ATRT-SHH tumors resemble mid/hindbrain progenitor cells, while intracranial as well as extracranial MYC tumors share genetic programs and potentially originate from fetal PGCs. We used a murine PGC-specific *Smarcb1* knockout model to validate PGCs as a cellular origin of RT. Furthermore, comparison analyses with PGCs uncovered an epigenetic imbalance between MYC tumors and PGCs, which is sustained by epigenetically-driven subclones and can be successfully targeted by the DNA demethylating agent decitabine.

## Results

### Smarcb1 deficiency in early embryonic Sox2-positive cells promotes RT development

Several mouse models have underlined the potential of *Smarcb1* as a tumor suppressor gene^16,18,25^. To identify the responsible RT precursor cells, we generated different GEMMs in which biallelic loss of *Smarcb1* is driven by constitutive cell-type specific expression of cre recombinase under the control of distinct promoters (*Nestin-cre::Smarcb1*^*fl/fl*^*, hGFAP-cre::Smarcb1*^*fl/fl*^*, Math1-cre::Smarcb1*^*fl/fl*^*, Sox2-cre::Smarcb1*^*fl/fl*^ and *Olig1-cre::Smarcb1*^*fl/fl*^*)*. These are active in early neural stem or progenitor cells with diverse temporal onsets of gene expression (see Supplementary Table 1 for details). *Nestin-cre*-, *Olig1-cre*- derived litters (Supplementary Fig. 1B, C) carrying homozygous loss of *Smarcb1* were not viable, and *hGFAP-cre::Smarcb1*^*fl/fl*^ mice had a short survival time (Supplementary Table 1). The analyses of *Nestin-cre::Smarcb1*^*fl/fl*^ and *Olig1-cre::Smarcb1*^*fl/fl*^ embryos at E14.5 disclosed Smarcb1-negative hyperproliferative areas in the hypothalamic region (Supplementary Fig. 1B, C). In *Nestin-cre::Smarcb1*^*fl/fl*^ mice, similar areas were also found within the cerebellar hemisphere (Supplementary Fig. 1B, e-f). The *Math1-cre::Smarcb1*^*fl/fl*^ model with long-term viable progeny exhibited severe motor defects and ataxia^26^. Collectively, none of these strains was prone to tumor development (Supplementary Table 1).

Subsequently, we generated heterozygous mouse strains, in which the second *Smarcb1* allele can be lost at a random time point during development. Whereas neither *Nestin-cre::Smarcb1*^*fl/+*^ nor *Olig1-cre::Smarcb1*^*fl/+*^ mouse strains showed any pathological phenotype (Supplementary Fig. 1Ba, Ca’, Supplementary Table 1), *hGFAP-cre::Smarcb1*^*fl/+*^ and *Math1-cre::Smarcb1*^*fl/+*^ mice gave rise to different tumor entities with a tumor penetrance of 11% and 4%, respectively. Tumors were further characterized by immunohistochemistry using markers such as Ki67, CD3, CD45, Hepar, Inhibin as well as H&E and PAS stainings. After evaluation of immunohistochemistry, localization, and morphology, tumors were assigned as most likely adenoma/adenocarcinoma, lymphoma or hepatocellular carcinoma (Supplementary Fig. 2A, B and Supplementary Table 1). These tumors presented loss of Smarcb1 expression, but lacked typical histological features of RT.

**Fig. 1:**
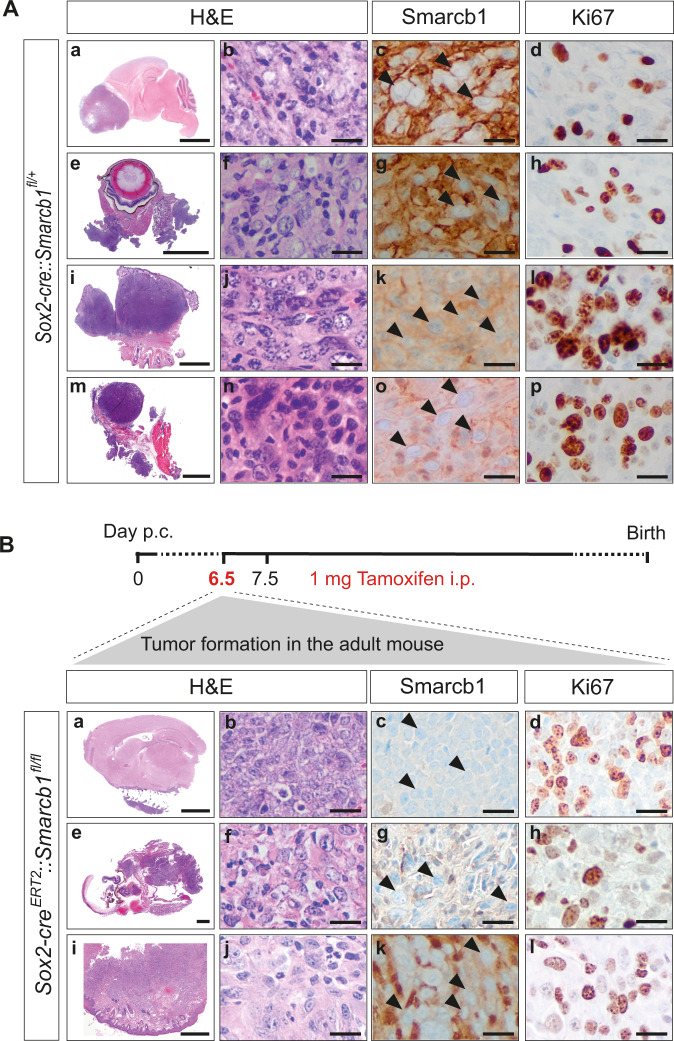
Smarcb1 deficiency in early embryonic Sox2-positive cells promotes RT development. **A** Representative H&E stainings of intra- and extracranial tumor samples derived from constitutive *Sox2-cre::Smarcb1*^*fl/+*^ mice. Tumors were detected in the frontal-basal region of the brain (**a**–**d**) or in the eye (**e**–**h**), and extracranial tumors (eRT) were localized in the oral cavity (**i**–**l**) or in the thorax (**m**–**p**). All tumors are characterized by loss of Smarcb1 protein (**c**, **g**, **k**, **o**) and are proliferative as indicated by Ki67 staining (**d**, **h**, **l**, **p**). Scale bars indicate 200 µm (**a**, **e**, **i**, **m**), and 20 µm in all remaining images. Arrowheads point at Smarcb1-negative cells. *n* = 19 tumors were stained, representative images are shown. **B** *Sox2-cre*^*ERT2*^*::Smarcb1*^*fl/fl*^ mice developed RT when loss of *Smarcb1* was induced *in utero* by the application of tamoxifen at E6.5 *post coitum (p.c.)* (upper scheme), but not later. Mice developed intracranial (IC) tumors at the lower brain surface (**a**–**d**), at the eye region (**e**–**h**) or eRT in the oral cavity (**i**–**l**). Smarcb1 staining depicts loss of Smarcb1 protein (**c**, **g**, **k**) and Ki67 staining indicates high proliferative capacity (**d**, **h**, **l**). Scale bars indicate 100 µm (**i**), 50 µm (**a**, **e**), and 20 µm in all remaining images. Arrowheads point at Smarcb1-negative cells. *n* = 14 tumors were analyzed, representative images are shown. See also Supplementary Figs. 1–3.

Exclusively *Sox2-cre::Smarcb1*^*fl/+*^ mice developed intra- and extracranial RT (Fig. 1A). Intracranial RT (IC-RT) and spinal tumors were found at various localizations of the CNS: supratentorial in the frontal cortex (Fig. 1A, a–d), eye/optic tract (Fig. 1A, e–h), basal side of the brain and spinal canal (Supplementary Table 2). Extracranial tumors (eRT) originated in the soft tissues of the head and neck region (Fig. 1A, i–l), or in the thorax (Fig. 1A, m–p). H&E staining confirmed the typical morphology of RT cells with large, prominent nuclei and eosinophilic cytoplasm (Fig. 1A, b, f, j, n). All samples exhibited a complete loss of Smarcb1 protein (Fig. 1A, c, g, k, o), and Ki67, a marker of cell proliferation, was robustly detected in these tumors, indicating a high proliferation capacity (Fig. 1A, d, h, l, p).

Since tumor penetrance was only 18% in this constitutive heterozygous *Sox2-cre* model (Supplementary Table 1) and a vulnerable time window of RT induction has already been described for ubiquitously inducible *Smarcb1* knockout models and for *Smarcb1* loss in neural crest cells^16,18^, we next assessed the susceptibility of tumor initiation in an inducible *Sox2-cre* system (further referred to as *Sox2-cre*^*ERT2*^). We established the *Sox2-cre*^*ERT2*^*::Smarcb1*^*fl/fl*^ knockout model, in which *Smarcb1* depletion was induced *in utero* by the application of a single dose of tamoxifen at E6.5. Tumor locations in these mice were comparable to those observed in *Sox2-cre::Smarcb1*^*fl/+*^ mice. Furthermore, histological examination confirmed histopathological features of human RT (Fig. 1B). Additionally, we observed a slightly increased tumor incidence (22%) and significantly decreased latency (average age of 27 weeks) in *Sox2-cre*^*ERT2*^ compared to *Sox2-cre* mice (average age of 41 weeks) (Supplementary Fig. 3A, Supplementary Table 1).

To further explore the overall low tumor penetrance, we utilized the previously described GEMM with conditional cre-driven inactivation of *Smarcb1* under the ubiquitous *Rosa26* promoter^16,27^. Direct comparison of both GEMMs revealed that *Rosa26-cre*^*ERT2*^ mice develop tumors earlier, at a median age of 17 weeks, and more frequently, with 40% penetrance (Supplementary Table 1). These observations might be a consequence of the distinct promoter specificities as well as recombination efficiency. Regarding the latter, cre recombination indeed only takes place in a few cells from both *Sox2-cre*^*ERT2*^, and *Rosa26-cre*^*ERT2*^ *Smarcb1* models as indicated by the occasional loss of Smarcb1 protein (Supplementary Fig. 3B). Further to note, we used low concentrations of tamoxifen for induction to avoid abortions. To precisely determine to which brain areas the subset of fully recombined E6.5 *Sox2*-positive, *Smarcb1*-negative cells localize, we crossed *Sox2-cre*^*ERT2*^*::Smarcb1*^*fl/fl*^ with a R26-stop-EYFP fate reporter line. Upon tamoxifen application at E6.5, all cells being *Sox2*-positive at the time point of recombination are labeled with an EYFP reporter, regardless of their *Sox2* expression at later stages of development. Immunofluorescence analysis of this fate mapping confirmed the highest EYFP expression at brain areas in which ATRT are detected in *Rosa26-cre*^*ERT2*^*::Smarcb1*^*fl/fl*^, thus harboring high tumorigenic potential (these regions are referred to as tumorigenic regions) (Supplementary Fig. 3C, D). Taken together, we conclude that selective *Smarcb1* abrogation in *Sox2*-positive early precursor cells at E6.5 is sufficient to drive RT formation in GEMMs.

### Murine tumors recapitulate features of human rhabdoid tumors

**Fig. 2:**
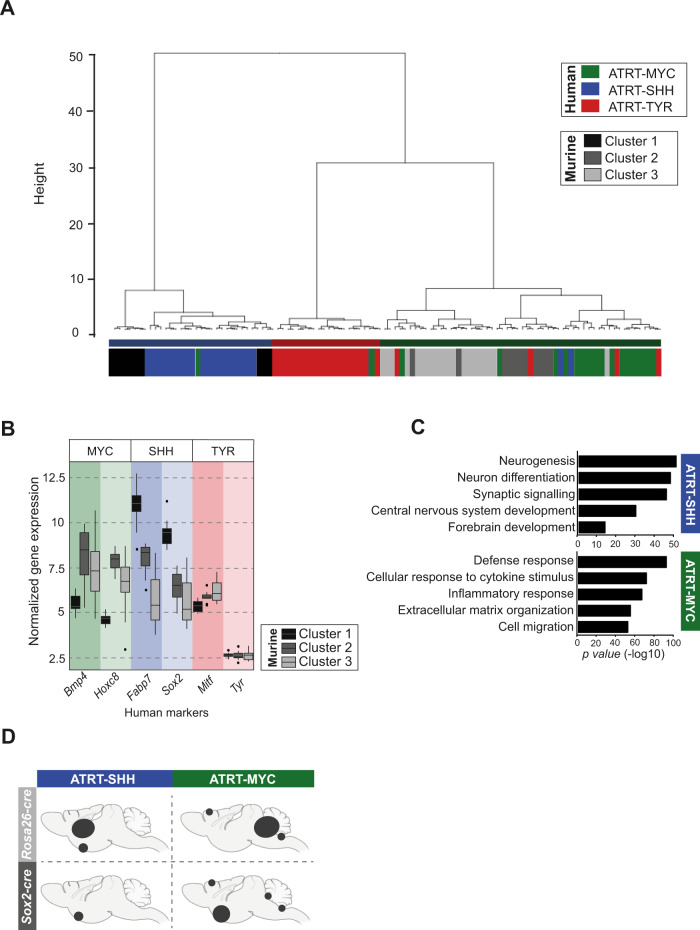
Murine tumors recapitulate features of human rhabdoid tumors. **A** Unsupervised hierarchical clustering of 67 human ATRT samples^7, 83^ and 41 murine RT samples derived from *Sox2-cre, Sox2-cre*^*ERT2*^ *Smarcb1* and *Rosa26-cre*^*ERT2*^*::Smarcb1*^*fl/fl*^ knockout models. Subgroup annotation for human tumors was derived from the original publication. Three clusters of murine samples were found by dimensionality reduction and clustering. **B** Boxplots display the expression of known marker genes of each RT human subgroup, in the three murine clusters (*n* = 41 biologically independent samples, grouped into cluster1: *n* = 10 samples; cluster2: *n* = 11 samples and cluster3: *n* = 20 samples). For each box, the lower and upper bounds represent the 25th and 75th percentiles; the center corresponds to the 50th percentile (median). The upper whisker extends to the largest value no further than 1.5 * IQR from the bound (where IQR is the interquartile range, or distance between the 25th and 75th percentiles). The lower whisker extends to the smallest value at most 1.5 * IQR of the bound. Data beyond the end of the whiskers are called outlying points and are plotted individually. **C** Gene ontology (GO) analysis of upregulated differentially expressed genes (DEGs) of murine ATRT-SHH *versus* ATRT-MYC samples. Only significantly enriched GO terms are shown (*x*-axis, −log10 scale). The R package limma^86^ was used for differential expression analysis (adjusted *p*-value by Benjamini & Hochberg method with threshold = 0.01; logFC threshold = ±2). Functional annotation was performed using ToppGene Suite^87^ (with statistical method as probability density function) considering GO terms enriched with an adjusted *p*-value (FDR) < 0.05. **D** Schematic representation indicates preferential brain areas of ATRT-SHH and ATRT-MYC tumors in *Sox2-cre, Sox2-cre*^*ERT2*^ (*n* = 18) and *Rosa26-cre*^*ERT2*^ *Smarcb1* (*n* = 9) knockout models (dot sizes depict the frequency of occurrence). See also Supplementary Figs. 4, 5, and source data file.

To assess whether murine tumors recapitulate human ATRT subgroups, we utilized gene expression profiling (GEP) of tumors derived from the *Sox2-cre, Sox2-cre*^*ERT2*^ and *Rosa26-cre*^*ERT2*^ *Smarcb1* knockout models. As a first step, we sought to identify molecular subgroups inside the murine dataset, composed of 24 IC-RT tumors (*n* = 8 from *Sox2-cre/Sox2-cre*^*ERT2*^ and *n* = 16 from *Rosa26-cre*^*ERT2*^ mice), 14 eRT tumors (*n* = 6 for *Rosa26-cre*^*ERT2*^, *n* = 8 for *Sox2*-cre/*Sox2-cre*^*ERT2*^ *Smarcb1* models) and 3 spinal RT tumors (*n* = 1 from *Sox2-cre/Sox2-cre*^*ERT2*^, *n* = 2 from *Rosa26-cre*^*ERT2*^) (Supplementary Table 2). Three clusters were defined by principal component analysis (PCA), followed by UMAP^28^ (Uniform Manifold Approximation and Projection) and hierarchical clustering (Supplementary Fig. 4A, B). As a second step, we integrated these murine tumors with 67 human ATRT samples published by Johann et al.^7^ to investigate transcriptional similarities between the two species. Through dimensionality reduction and clustering, we observed that murine cluster 1 was grouped together with human ATRT-SHH samples, while both murine clusters 2 and 3 had highest similarity with human ATRT-MYC (Fig. 2A and Supplementary Fig. 4C). None of these murine samples clustered to the human ATRT-TYR subgroup. IC-RT of *Rosa26-cre*^*ERT2*^*::Smarcb1*^*fl/fl*^ mice as well as mice of the Sox2-cre models fall either into the human ATRT-MYC or into the human ATRT-SHH (*Rosa26-cre*^*ERT2*^*::Smarcb1*^*fl/fl*^: ATRT-MYC (*n* = 8) and SHH subgroup (*n* = 8); *Sox2-cre* models: ATRT-MYC subgroup (*n* = 6) and ATRT-SHH (*n* = 2)) (Supplementary Fig. 4B, D). All murine eRT samples, as well as spinal samples, clustered exclusively to the ATRT-MYC subgroup (Fig. 2A and Supplementary Fig. 4B). Additionally, we collected known markers from the literature used by Johann et al.^7^ and Ho et al.^13^ to demonstrate differences between the three RT subgroups and plotted their expression in our three murine clusters. As expected, murine cluster 1 showed higher expression of ATRT-SHH markers (e.g., *Sox2* or *Fabp7)*, while clusters 2 and 3 had a similar high expression of ATRT-MYC specific markers (e.g., *Bmp4* or *Hox8*) (Fig. 2B and Supplementary Fig. 4E).

To investigate differences between murine ATRT-MYC and ATRT-SHH tumors, differentially expressed genes (DEGs) were analyzed, independently of the genetic mouse background. While gene ontology (GO) analysis of DEGs upregulated in murine ATRT-SHH revealed an enrichment of processes related to neural development, ATRT-MYC upregulated genes were related to immune system response or cell migration (Fig. 2C). This is in agreement with murine bulk gene expression data from previously published studies^16^.

Murine ATRT-MYC, spinal RT, and eRT presented similar up- and downregulated genes in comparison to ATRT-SHH, as depicted in Supplementary Fig. 4F.

We did not observe significant differences regarding infra- and supratentorial distributions of ATRT tumors between both subgroups in the model systems. Furthermore, both tumor subgroups shared some localizations within the brain (basal area). Nonetheless, subgroup-specific areas of tumor occurrence were also observed: for instance, only ATRT-MYC tumors arose lateral of the cerebellum or at the trigeminal nerve or the eye, whereas all tumors found next to the subventricular zone belonged to the ATRT-SHH subgroup (Fig. 2D). Overall, these results match with the described predominant tumor locations of distinct molecular subgroups in human ATRT^29^.

Even though both ATRT-SHH and MYC tumors arise from *Smarcb1*-negative *Sox2*-positive precursor cells, *Sox2* expression levels in the tumor itself vary between the tumor subgroups of both murine and human samples (Fig. 2B and Supplementary Fig. 5A, B).

### RT of different localisations share repetitive transcriptional programs

**Fig. 3:**
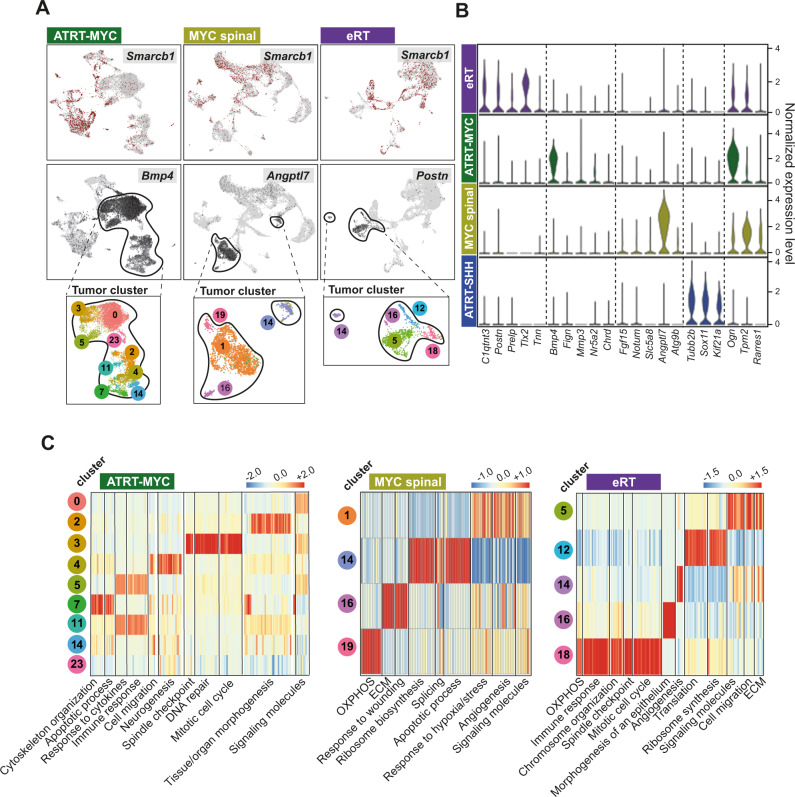
RT of different localizations share repetitive transcriptional programs. **A** UMAP plots show clustering of murine MYC tumor single-cell transcriptomes subdivided into ATRT-MYC (left, *n* = 5 samples), MYC tumors from the spinal canal (middle, referred to as MYC spinal, *n* = 3 samples) and eRT (right, *n* = 3 samples). *Smarcb1*-negative tumor cells (upper row) and expression of the exemplary MYC subtype-specific genes depict tumor clusters (TCs) (circled clusters, TC numbers are indicated in magnified insets). **B** Violin plots depict gene expressions of top-ranked localization-specific tumor markers derived from DE analysis comparing only tumor cells of all three MYC subtypes. **C** Heatmaps show the upregulated expression of functional gene classes across individual TCs of the three MYC subtypes. See also Supplementary Fig. 6 and source data file.

We aimed to better understand the heterogeneity and cellular origin of RT. For this purpose, we performed scRNA-seq analyses from murine tumor material (five ATRT-MYC, three MYC spinal, three eRT samples and two ATRT-SHH; Supplementary Fig. 12 and Supplementary Table 3). After pre-processing and quality control, retained cells were embedded into different UMAP plots according to the tumor subtypes. First, *Smarcb1* expression was used to distinguish *Smarcb1*-negative tumor cells from tumor-associated stroma cells (Fig. 3A, upper rows and Supplementary Fig. 6A). We confirmed that *Smarcb1*-negative cells had good quality by plotting the total number of genes per cell (Supplementary Fig. 6B). By comparing gene expression of *Smarcb1*-negative tumor clusters (TCs) of all three MYC subtypes, we identified localization-specific tumor markers. For instance, *Bmp4* is specific for ATRT-MYC neoplastic cells, *Angptl7* characterizes spinal tumor cells and *Postn* is strongly expressed by eRT cells (TCs are depicted in insets for each subtype) (Fig. 3A, B). In addition, we defined genes being expressed by all three MYC tumor groups, but absent in SHH tumors (e.g., *Ogn, Tpm2*, and *Rarres1*). Vice versa, typical SHH marker genes are not expressed by tumor cells of all three MYC subtypes (Fig. 3B).

Using differential expression (DE) analysis between TCs of each subtype, intratumoral heterogeneity becomes evident by distinct cluster-specific biological processes. For example, ATRT-MYC TC3 is mostly enriched in genes related to spindle checkpoint, DNA repair and mitotic cell cycle, whereas TC2 shows upregulation of genes involved in tissue morphogenesis (Fig. 3C). Even though MYC spinal and eRT are composed of fewer TCs compared to ATRT-MYC samples, their TCs show similar enrichment of related gene functions (Fig. 3C). Despite these intratumoral differences as well as minor intertumoral heterogeneity (Supplementary Fig. 6C), there is a considerable overlap of biological processes shared by all MYC subtypes, which could imply a common cellular origin.

In summary, using single-cell technology we unraveled murine MYC tumor cell heterogeneity and identified similar gene expression patterns between MYC tumors from different localizations.

### Murine SHH and MYC tumors arise from distinct cells of origin

**Fig. 4:**
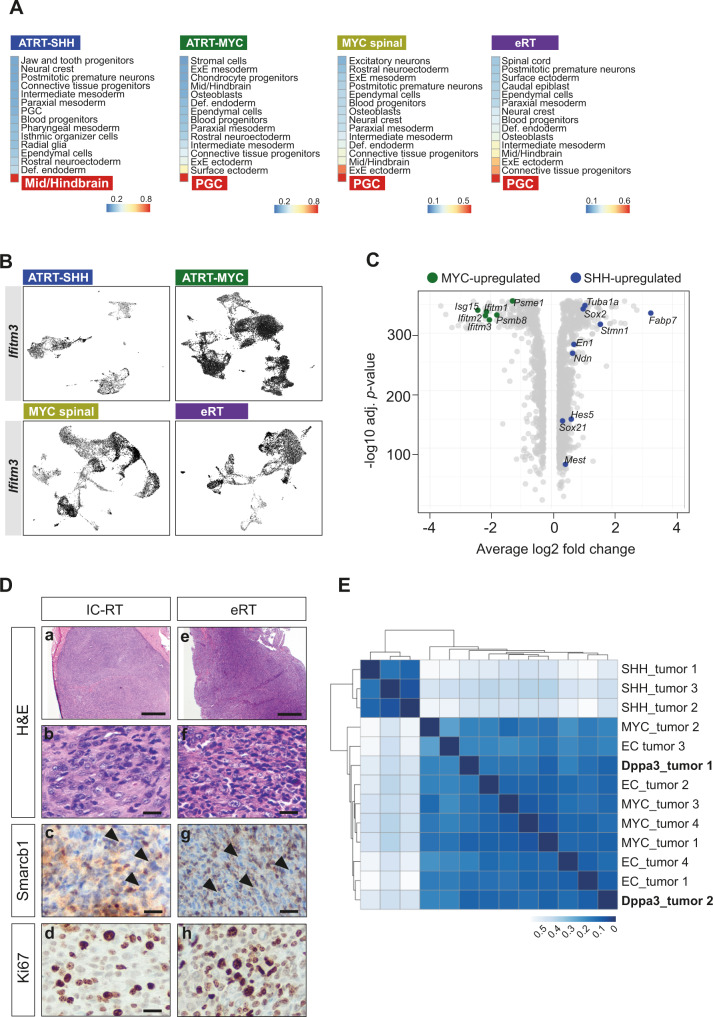
Murine SHH and MYC tumors arise from distinct cells of origin. **A** Similarity scores of different murine RT subtypes and embryonal cell types as calculated by logistic regression. Colors represent the probability of high (red) to low (blue) similarity. Complete results with scores at the single-cell level are shown in Supplementary Fig. 8. **B** UMAP plots of *Ifitm3*-expressing cells in ATRT-SHH and MYC tumors of all three subtypes. **C** Volcano plot of up- (in ATRT-SHH) and downregulated (upregulated in ATRT-MYC) DEGs derived from the comparison between SHH and MYC tumor cells. Differential expression analysis was computed on the unintegrated data using MAST algorithm^88^. Statistically significant genes were considered having a *q*-value < 0.05 (Bonferroni correction). **D** Representative H&E stainings of intra- (*n* = 1) and extracranial (*n* = 1) tumor samples derived from *Dpp3a-cre::Smarcb1*^*fl/+*^ mice. Tumors were detected adjacent to the trigeminal nerve (**a**–**d**) or in the maxillary region (**e**–**h**). Both tumors present loss of Smarcb1 protein (**c**, **g**) and are highly proliferative, as indicated by Ki67 staining (**d**, **h**). Scale bars indicate 500 µm (**a**, **e**), and 20 µm in all remaining images. Arrowheads point at Smarcb1-negative cells. **E** Heatmap representing the inter-sample distances calculated between two murine samples derived from the *Dpp3a-cre::Smarcb1*^*fl/+*^ model and published murine RTs of the SHH, MYC and extracranial subgroups (GSE137633). Distances between samples are calculated as 1-correlation, while the hierarchical clustering uses Ward.D2 method. Only a subset of genes corresponding to known subgroup markers was considered prior to the distance calculation. See also Supplementary Figs. 7–10. Source data are provided as a Source data file.

Using our GEMMs, we exposed that *Smarcb1* abrogation at E6.5 in *Sox2*-positive cells leads to RT formation in adult mice. As we sought to explore the precise cellular identity of RT, we took advantage of available single-cell transcriptome atlases of murine embryos between E6.5 and E13.5^30,31^, which harbor the complete cell diversity and cover the vulnerable developmental stages of RT transformation. For each of the two atlases, we randomly sampled 50,000 cells, transferred cell type labels as annotated in the original studies and performed dataset integration using Seurat^32^. By doing so, we made sure that all developmental cell types were recapitulated (Supplementary Fig. 7A) and a temporal transition of cells from early to later embryonic time points was kept in the resulting UMAPs (Supplementary Fig. 7B). While *Sox2* is broadly expressed at E6.5, its expression is restricted to certain cell types afterward (Supplementary Fig. 7C, D). To investigate the similarity between tumor cells and each cell type of the integrated embryo atlas, we used a logistic regression approach that had been shown previously to successfully recapitulate the cell of origin (COO) of renal tumors^33^. This algorithm uses gene expression patterns of each embryonic cell type to build a logistic function and then predicts a score of similarity between the given reference cell type and the tumor cells. We only considered tumor cells (neglecting stroma cells) and performed individual analysis per subtype. Strikingly, we obtained mid/hindbrain progenitor cells as most similar embryonic cell type for ATRT-SHH tumor cells, whereas primordial germ cells (PGCs) emerged as the cell type with the highest similarity score for ATRT-MYC (MYC IC-RT and MYC spinal tumors) and eRT (Fig. 4A, Supplementary Fig. 8). Functional gene network analysis revealed that subsets of genes that are found by the logistic functions as predictor genes are tightly connected (Supplementary Fig. 9A, B). Focusing on mid/hindbrain predictors, we noticed that *Shh* itself plays a central role in this gene network, being interconnected with different members of the SRY-related HMG-box (SOX) family of transcription factors (*Sox2*, *Sox9* and *Sox21*), *Fgf* genes and *Fabp7* (Supplementary Fig. 9A). This might further corroborate the similarity between tumor cells of the SHH subgroup and mid/hindbrain precursor cells.

In contrast, the PGC predictor gene network consists of genes such as *Ifitm3*, different components of the proteasome complex (*Psmb8* or *Psme1*) and genes involved in interferon signaling (*Isg15* or *Igtp*) (Supplementary Fig. 9B). For instance, we examined the expression of *Ifitm3* in our four single-cell tumor datasets and show that this gene is strongly expressed by all MYC subtypes (Fig. 4B). *Ifitm3* is also detected in cells of the ATRT-SHH subgroup, albeit to significantly lower levels. Interestingly, when directly comparing SHH *versus* MYC tumor cells via DE analysis, we found some of these mid/hindbrain and PGCs predictor genes as significantly upregulated in the respective subgroups (Fig. 4C). Finally, we evaluated the expression of predictor genes that showed highest expression in our murine single-cell tumor cells in two independent cohorts of human ATRT samples, for which bulk gene expression data was available^7,34^. Unexpectedly, by using this subset of predictor genes for PGC and mid/hindbrain, unsupervised hierarchical clustering reached high classification accuracy for both datasets (SHH: ~72% and ~82%; MYC: ~79% and 75%, respectively for Torchia et al.^34^ and Johann et al.^7^ datasets), implying that these genes have expression patterns that are highly specific for ATRT-SHH and ATRT-MYC subgroups (Supplementary Fig. 9C, D).

To biologically validate the COO of MYC-RT, we used a mouse model with loss of *Smarcb1* in PGCs (Dppa3-cre; Shanghai Model Organisms #NM-KI-00040). While homozygous *Dppa3-cre::Smarcb1*^*Fl/Fl*^ mice showed embryonic lethality, heterozygous *Dppa3-cre::Smarcb1*^*Fl/+*^ mice developed intracranial and extracranial RT (*n* = 3) (Fig. 4D). We performed bulk RNAseq of two RT generated in *Dppa3-cre::Smarcb1*^*Fl/+*^ mice and confirmed by unsupervised hierarchical clustering with murine RT (retrieved from GEO: GSE137633), that (i) these murine tumors cluster together with the RT-MYC subgroup (Fig. 4E) and (ii) they have comparable expressions of RT subgroup markers (Supplementary Fig. 10A–C).

In summary, based on computational predictions and by using cell specific *Smarcb1* knockout mouse models we unraveled different potential COO for murine SHH and MYC tumors. We identified *Sox2*-positive PGCs as one progenitor cells for intra- and extracranial MYC RT subtypes.

### RT of the MYC subgroup have a deregulated transcriptome and epigenome compared to PGCs

**Fig. 5:**
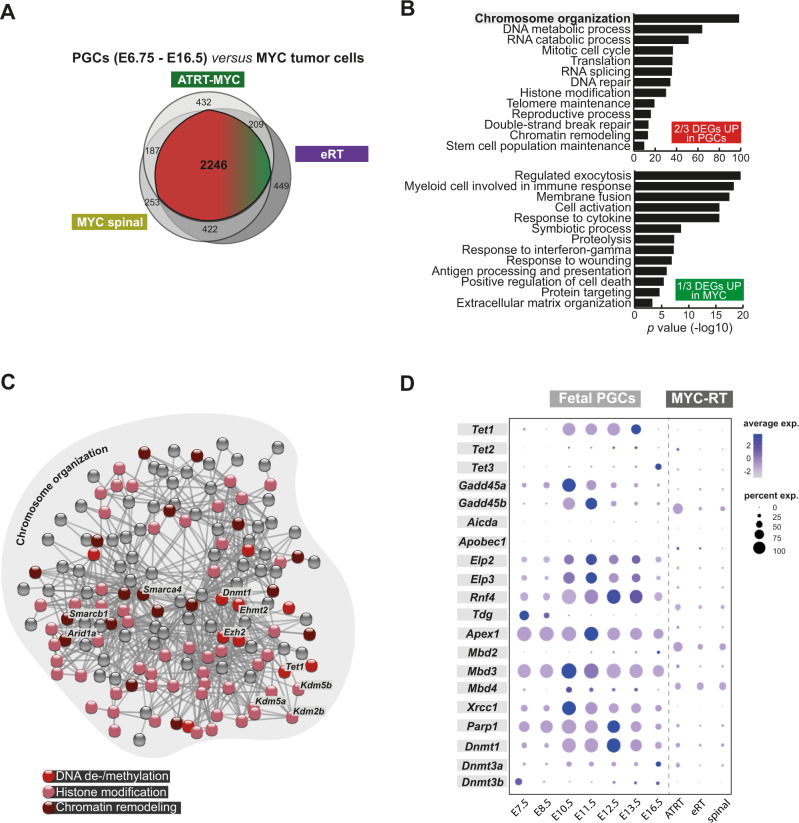
RT of the MYC subgroup have a deregulated transcriptome and epigenome compared to PGCs. **A** Venn diagram showing the overlap of DEGs derived from the comparison between PGCs (E6.75–E16.5)^30,35^ and MYC tumor cells of each subtype. Two-thirds of overlapping DEGs are upregulated (in PGCs, in red) and one-third are downregulated (upregulated in tumor cells, in green). **B** GO term analysis of the overlapping DEGs between PGCs and MYC tumor cells among all subtypes. Only significantly enriched GO terms were considered (*x*-axis, −log10 scale). Differential expression analysis used MAST algorithm^88^. Statistically significant genes were considered having a *q*-value < 0.05 (Bonferroni correction). Functional annotation was performed using ToppGene Suite^87^ (with statistical method as probability density function) considering GO terms enriched with an adjusted *p*-value (FDR) < 0.05. **C** STRING interaction network of shared upregulated DEGs (upregulated by PGCs) related to the GO term annotation ‘chromosome organization’. Each dot represents one gene. Major cellular functions are indicated by different colors and exemplary genes are highlighted. **D** Dotplot representing the expression of selected epigenetic-related genes in fetal PGCs (E7.5–E11.5) and the three MYC subtypes (MYC spinal, eRT and ATRT-MYC). See also Supplementary Fig. 11 and source data file.

To investigate the molecular framework underlying a potential PGC transformation into MYC tumor cells, we compared single cell transcriptomes of the latter with PGCs from various developmental stages between E6.75 and E16.5 (in total 15,106 single cells^30,35^), and performed DE analysis. A total number of 2246 overlapping DEGs between all three MYC subtypes indicates a high degree of shared expression patterns among MYC tumors irrespective of their body location (Fig. 5A). One-third of these overlapping genes are upregulated in MYC tumor cells and are related to GO terms such as regulated exocytosis, response to cytokines or interferon-gamma, and antigen presentation (Fig. 5B). This observation is in line with recently published studies^12,27,36^, pointing toward a strong immune cell influence in RT of the MYC subgroup. In contrast, two-thirds of overlapping DEGs are upregulated in PGCs and can be linked to biological functions, which are also observed in the lifecycle of PGCs (e.g. DNA repair, mitotic cell cycle or RNA catabolic processes^35^) (Fig. 5B). The GO term ‘chromosome organization’ was most significantly enriched suggesting that this process strongly distinguishes genuine PGCs from developed tumor cells. Involved genes form a highly interconnected functional network, including *Smarcb1* and other SWI/SNF subunits, and can be related to processes linked to histone modifications, DNA methylation, and demethylation (Fig. 5C). This result implies that deregulated epigenetic processes might be involved in the cellular transformation of PGCs to RT.

Although it has been previously described that human mitotic PGC stages are relatively homogeneous^22^, there are noteworthy differences before the onset of sex determination^35,37^. Exemplarily, PGCs harbor a dynamic methylome undergoing excessive DNA demethylation waves during embryogenesis^37–40^. We performed trajectory analysis using single-cell transcriptomic datasets from PGCs ranging from E7.5 to E16.5^30,35^ to reconstruct PGC differentiation along a pseudotime-ordered axis. This trajectory splits into two branches based on male and female sex specification at the later time points (Supplementary Fig. 11A). Selected genes classified into distinct PGC-specific biological functions exhibit a transition of expression along pseudotime-ordered PGCs (Supplementary Fig. 11B). In detail, early mitotic PGCs (marked by mitotic cell cycle genes, e.g., *Ccnb1* or *Ccna2*) express pluripotency genes (*Pou5f1* or *Lefty1*) and are characterized by a migratory phenotype (*Dnd1*, *Ifitm1*, or *Gjb3*). In addition to DNA demethylation, germ cell reprogramming involves histone modifications through histone demethylases such as *Kdm5a* or methyltransferases, e.g. *Prmt1* or *Suv39h1*. At later developmental stages, when PGCs have completed directed migration and have entered the gonads, sex specification takes place and female (*Filga*-positive) cells enter meiosis network (e.g., *Stra8*) (Supplementary Fig. 11B). Interestingly, *Dnd1*, an embryonal PGC-specific marker controlling PGC fate maintenance by inhibiting somatic gene expression^41,42^, is not expressed by MYC tumor cells, suggesting that its loss might play a central role in the initiation of cellular transformation and misguidance of these cells to distant anatomic localizations^43^ (Supplementary Fig. 11C, D). Moreover, many epigenetic related genes, directly or indirectly involved in the regulation of DNA methylation/demethylation mechanisms^44^, show a dynamic regulation along the developmental stages of fetal PGCs, which is in concordance with the already described PGC-specific demethylation wave phenomenon^37^. All three MYC tumor clusters present a comparable expression of those genes among them, and a lower expression compared to their hypothesized cells of origin (Fig. 5D).

Taken together, due to the salient incongruity between fetal PGCs and MYC tumor cells, which is most likely based on the highly dynamic epigenome of PGCs, hereinafter we aimed to gain deeper insights into the deregulated transcriptomes of murine RT.

### Epigenetically active subpopulations raise cellular heterogeneity in MYC tumor cells

Perturbations of *Smarcb1* as a core component of the SWI/SNF chromatin remodeling complex are directly linked to critical cellular processes including cell cycle progression, development and cell differentiation^45^. Moreover, the cell cycle regulatory machinery has been investigated in the context of reprogramming and (trans-)differentiation as it impacts chromosome architecture, epigenome and consequently transcriptional programs, also with respect to PGC transition^46,47^. Thus, we hypothesized that epigenetically defined subclones contribute to tumor cell heterogeneity in RT and investigated these functional correlations in tumor single-cell transcriptomes.

**Fig. 6:**
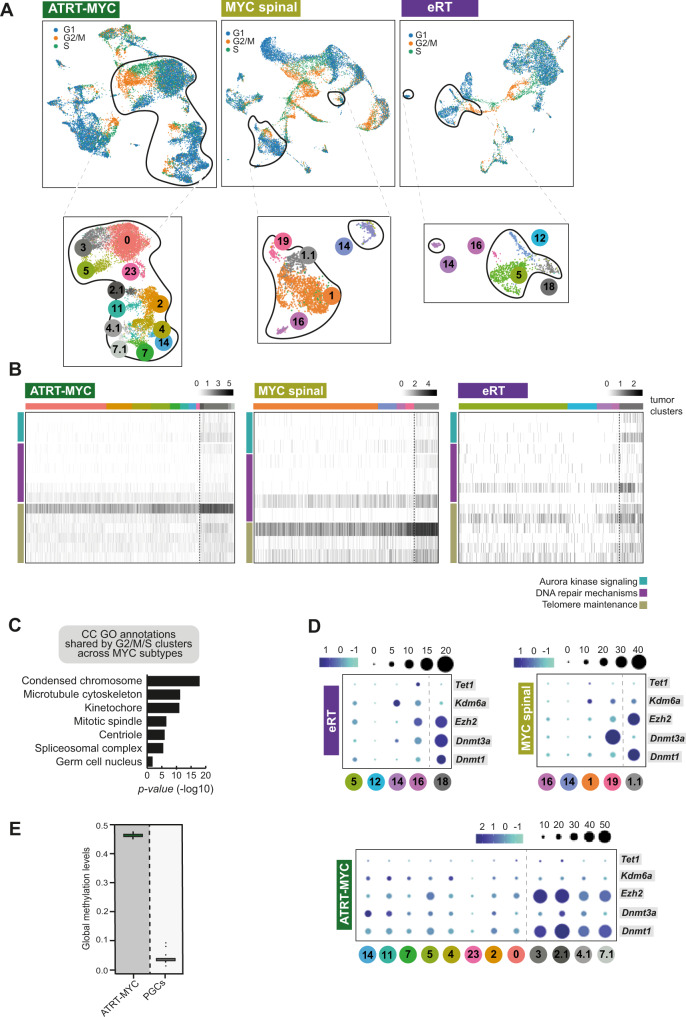
Epigenetically active subpopulations raise cellular heterogeneity in MYC tumor cells. **A** UMAP indicates the expression of 98 cell cycle-related genes^48^ in RT tumors from different MYC subtypes. Zoom-in boxes depict TCs with newly added subclusters to highlight cells in G2/M or S phase (subclusters in gray are further referred to as mitotic cells). **B** Heatmaps show shared biological processes of TCs in G2/M or S phase across MYC subtypes. **C** GO term analysis of shared upregulated DEGs (upregulated in mitotic TCs) derived from the comparison between mitotic tumor cells and tumor cells expressing G1 phase-specific genes. Only significantly enriched cellular component (CC) terms are shown (*x*-axis, −log10 scale). Differential expression analysis used MAST algorithm^88^. Statistically significant genes were considered having a *q*-value <0.05 (Bonferroni correction). Functional annotation was performed using ToppGene Suite^87^ (with statistical method as probability density function) considering GO terms enriched with an adjusted *p*-value (FDR) < 0.05. **D** Dot plots depict the relative gene expression of distinct epigenetic modifiers derived from a direct comparison between TCs of each MYC subtype. The fraction of cells (black dots) and the average scaled expression (blue scales) are shown. **E** Global methylation levels are shown for PGCs (*n* = 13 biologically independent samples, ranging week7-week19) and ATRT-MYC samples (*n* = 33 biologically independent samples). For each box, the lower and upper bounds represent the 25th and 75th percentiles; the center corresponds to the 50th percentile (median). The upper whisker extends to the largest value no further than 1.5 * IQR from the bound (where IQR is the interquartile range, or distance between the 25th and 75th percentiles). The lower whisker extends to the smallest value at most 1.5 * IQR of the bound. Data beyond the end of the whiskers are called outlying points and are plotted individually. Source data are provided as a Source data file.

Using a set of 98 cell cycle-related genes^48^, we were able to assign distinct cell cycle phases and visualize them in the UMAPs of MYC tumor subtypes (Fig. 6A). Interestingly, while most of the tumor cells are in G1 phase, we also detected tumor (sub-) clusters expressing G2/M and S phase-related genes (these TCs were further sub-clustered and colored in different shades of gray, see Fig. 6A, zoom-in boxes). A direct DE comparison between all TCs cycling through S or G2/M phase and TCs in the G1 phase showed a common upregulation of genes related to aurora signaling, DNA repair and telomere maintenance in all three MYC subtypes (Fig. 6B). Furthermore, a subset of these shared upregulated DEGs can be linked to chromosome condensation (Fig. 6C). Interestingly, when considering only tumor clusters, epigenetic modifiers like *Dnmt3a*, *Dnmt1* and *Ezh2*, indicative for methylated and inactive chromatin, are specifically expressed by mitotically active tumor cells (gray TCs in Fig. 6D). These results add information to published studies, which pointed out strong DNA methylation in RT^12^, by demonstrating the existence of epigenetically-driven subpopulations. DNA demethylation is catalyzed by active demethylases (e.g., *Tet1)* and other genes being involved in DNA demethylation (e.g., Mbd genes)^37,49,50^. While these genes are strongly expressed in early PGCs to ensure a hypomethylated epigenome, their expression is downregulated or largely abrogated in MYC tumor subtypes suggesting a deficiency of active demethylation (Fig. 5D). This, added to the fact that methylating enzymes like Dnmt1 or Dnmt3a are in proportion upregulated, tips the scales toward a global DNA hypermethylation in MYC RT (Fig. 6E).

Taken together, these analyses indicate that epigenetic mechanisms are deregulated in MYC RT in comparison to their COO, which is sustained by a subset of epigenetically-driven tumor cells.

### Counteracting epigenetic imbalance in rhabdoid tumors through targeted therapy

**Fig. 7:**
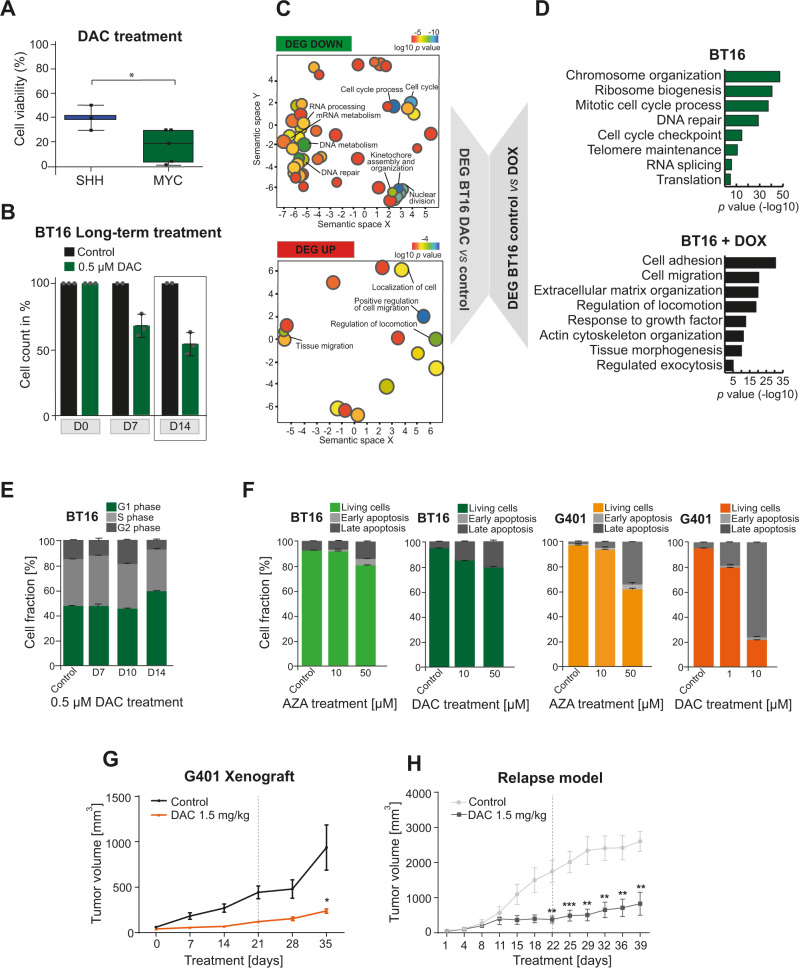
Counteracting epigenetic imbalance in MYC tumors through targeted therapy. **A** Eight human RT cell lines (3 for SHH and 5 for MYC subgroups) was tested for viability after 6 days decitabine (DAC) treatment (1 µM). A remarkable decrease in cell viability was observed in both subgroups, though the effect on MYC cells was more pronounced. (Unpaired-t-test, two-tailed, *p = 0.064). For each box, the lower and upper bounds represent the 25th and 75th percentiles; the center corresponds to the 50th percentile (median). The whiskers go down to the smallest value and up to the largest. **B** Graph depicts the cell number of DAC-treated BT16 (ATRT-MYC) cells over time. *n* = 2/3 technical replicates. Mean ± SD is shown; gray dots indicate individual values. **C** Overrepresented functional categories of up- and downregulated DEGs of DAC-treated BT16 cells over 14 days compared to untreated cells, further subjected to microarray profiling. Functional categories are clustered and visualized based on similarity using REVIGO^99^. Size and color of circles represents the *p* values (log10 scale) derived from ToppGene^87^ (statistical method as probability density function) considering terms enriched with an adjusted *p*-value (FDR) < 0.05. **D** GO term analysis of DEGs between BT16 cells before and after *SMARCB1* re-expression (BT16 + DOX) (data derived from Wang et al.^59^). Differential expression analysis was conducted with the R package DESeq2^95^. Only genes with adjusted p-value < 0.05 were considered (Benjamini–Hochberg correction). Functional annotation was performed as in (**C**). Only significantly enriched GO terms were considered (*x*-axis, −log10 scale). **E** Stacked bar graph depicts cell cycle analysis of BT16 cells treated with vehicle or DAC over time. Analysis based on *n* = 2/3 technical replicates, mean ± SD is shown. **F** Stacked bar graphs showing apoptosis assay results of BT16 or G401 tumor cells treated with DAC or AZA (5-azacytidine) over 7 days. *n* = 3 technical replicates, mean ± SD is shown. Tumor volume curve of **G** NOD/SCID mice (engraftment of G401 cells) treated with DAC (*n* = 6) or vehicle (*n* = 8) (Unpaired, two-tailed *T*-Test; *p = 0.0258) and **H** of a PDX model of a relapsed eRT treated with vehicle (*n* = 7) or DAC (*n* = 7) (Unpaired multiple *T*-test; **p < 0.001, ***p < 0.001). See also source data file.

As the SWI/SNF complex directly interacts with epigenetic modifiers possessing enzymatic activity, these modifiers are an attractive target in RT^51–55^. As (i) MYC tumors express lower levels of active DNA demethylases as well as other genes being involved in DNA demethylation, and (ii) DNMTs are upregulated in RT^56,57^, we hypothesize that their inhibition impacts tumor outcome through counterbalancing the epigenetic status of these tumors. As proof of principle, we performed cell viability assays using 3 human SHH cell lines and 5 human MYC cells lines treated with decitabine (DAC), a DNA-demethylating agent. We observed a decrease in cell viability to 40% and 20% in SHH and MYC, respectively, indicating that DAC exerts an effect on both RT subgroups (Fig. 7A). After having proved the efficacy, we conducted further in vitro assays by incubating cells of one ATRT-MYC cell line (BT16) and one extracranially-derived RT cell line (G401) in the presence of either DAC or 5-azacytidine (AZA), both selective and potent DNMT inhibitors^58^. To assess if inhibition of DNMTs blocks proliferation of RT cells, we performed a long-term treatment approach with 0.5 µM DAC over 14 days, which resulted in a remarkable decrease of BT16 cell number in comparison to the control (Fig. 7B). Further, gene expression profiling of these 0.5 µM DAC long-term treated BT16 cells indicated an upregulation of genes solely related to the regulation of cell migration and a downregulation of genes involved in cell cycle process, DNA metabolism and DNA repair (Fig. 7C). DE analysis of published RNA expression data of BT16 cells before and after SMARCB1 re-expression^59^ revealed an enrichment of genes related to comparable biological processes as seen upon DAC treatment (Fig. 7D), suggesting that DAC treatment would have comparable effects on gene transcription level as *Smarcb1* re-expression in ATRT-MYC cells. Cell cycle analysis after 14 days of DAC-treated BT16 cells revealed an increase of G1 phase cells (Fig. 7E). In addition, suppression of tumor proliferation was accompanied by an increase in apoptotic cells in both cell lines as demonstrated by the use of two different DNMT inhibitors (DAC and AZA) (Fig. 7F).

Next, we investigated the antitumoral activity in an in vivo xenograft model. Extracranially-derived G401 cells were engrafted into the flank of NOD/SCID mice. When tumors reached a defined size, mice were randomly segregated into untreated control (*n* = 8) and DAC-treated (1.5 mg/kg) cohorts (*n* = 6). Indeed, tumor volume was significantly reduced in treated *versus* control mice (*p = 0.0258, Fig. 7G). Based on the presumption that the genetic landscape of RT at relapse is very similar to the situation at diagnosis, epigenetic events rather than genetic selection could be leading causes of drug resistance. Hence, we wondered whether specifically targeting these epigenetic subclones could lead to equally favorable treatment outcomes as in primary tumors. Thus, we established a patient-derived xenograft model of a relapsed eRT transplanted into the flank of NOD/SCID mice. After engraftment, we followed a comparable DAC treatment scheme as reported above and observed significantly reduced tumor growth in DAC-treated (*n* = 7) *versus* control (*n* = 7) mice (Fig. 7H). Taken together, we confirm in vitro and in vivo efficacy of epigenetic drug treatments counteracting DNMT upregulated functions in RT and provide evidence of a potential relation of targeted therapy to the COO of MYC tumors.

## Discussion

RT are genetically and histologically well characterized, but little has been known about the intratumoral heterogeneity and the COO of these tumors is still a matter of debate^16,18,19,45,60^. It is proposed that ATRT-SHH arise from neural progenitors, for instance mid/hindbrain progenitors, while MYC RT and ATRT-TYR are suggested to derive from e.g., neural crest cells or other mesenchymal cell types^16–19,34,60^. Here, we demonstrate that induction of *Smarcb1* loss in *Sox2*-positive cells at E6.5 results in RT development of MYC and SHH subgroups. On the contrary, loss of *Smarcb1* in *hGFAP*- or *Math1*-positive precursor cells either resulted in severe brain malformations^26^ or gave rise to tumor entities distinct from RT. Interestingly, loss of *Smarcb1* in *Nestin-* or *Olig1*-positive precursor cells resulted in hyperproliferative, *Smarcb1*-negative lesions. These lesions might be RT pre-lesions, which cannot fully form RT, as these mice have an embryonal lethal phenotype. Attempts to rescue the lethality in *Smarcb1* depleted *Nestin-cre* mice were already described, but failed as *Smarcb1* expression in *Nestin*-positive progenitors seems to be indispensable for embryonic development^61^. Using constitutive and conditional *Smarcb1* knockouts in *Sox2*-positive cells, our data show that the time point and the specific cell population of *Smarcb1* loss determines whether a cell undergoes transformation or harbors an impaired proliferative capacity. This is consistent with published RT mouse models using different promoters to induce cre-mediated *Smarcb1* knockout, indicating that a specific stage in differentiation is essential for RT genesis^16,18^.

eRT, occurring at almost any site of the body, are similar to ATRT-MYC tumors and can show histological features of different lineages within the same tumor^6^. This and the suspected prenatal origin of these tumors imply a common cellular origin of intra- and extracranial MYC tumors harboring multipotent characteristics. In contrast, ATRT of the SHH or TYR subgroup are restricted to distinct areas of the brain^12^, which suggests a more differentiated, less plastic COO. Recently, Jessa et al. stated that group 2a/b (TYR/MYC) most probably originate outside the neuroectoderm, whereas group 1/SHH genes were detected in neuroectoderm, spinal cord and fore/midbrain progenitors of murine embryos^60^. Vitte et al.^18^ as well as Custers and colleagues^19^ suggest neural crest cells as the origin of MYC tumors.

Here, we utilized an unbiased, computational approach comparing murine single-cell transcriptomes and embryo reference atlases^30,31^ covering all cell types from vulnerable developmental stages, including different *Sox2*-expressing cell populations. We unraveled distinct potential COO for murine SHH and MYC subgroup tumors. While mid/hindbrain progenitor cells were identified as precursors for ATRT-SHH tumors, PGCs were discovered as COO of intra- and extracranial tumors of the MYC subgroup. However, we are aware that these bioinformatic algorithms “only” provide a prediction of the similarities of gene expression patterns. These approaches, for example, do not consider that during the tumor transformation process, specifically when epigenetic modifiers such as Smarcb1 are altered, the transcriptome of a cell might be significantly changed, thus leading to wrong predictions. Therefore, a biological validation was necessary and we used *Dppa3-cre::Smarcb1*^*Fl/+*^ mice to show that PGCs are one pool for the COO of MYC RT. Nevertheless, to circumvent possible experimental limitations in the biological validation, we suggest that further investigation i.e using different cell-specific knockout mouse models, will help to corroborate this finding. *Sox2* is expressed by both embryonic cell progenitors; however, it might not act as a cell-type defining gene. *Sox2* is functioning as a pluripotency gene in PGCs but is lost upon the first epigenetic reprogramming wave around E11.5 during embryogenesis^37^. Thus, based on the findings from our GEMMs, complete *Smarcb1* deletion in PGCs most likely occurs in the *Sox2*-expressing cell pool before E11.5 during their migration. If normal PGC development is disturbed, their fate is not preserved and cells become prone for somatic transdifferentiation^41^, hence *Sox2* may not be maintained in developed MYC tumors. A potential explanation are epigenetic mechanisms that alter the expression of distinct genes, such as *Sox2*, during RT transformation. Such epigenetically activated changes may occur only after tumor initiation, as the prediction of the COO was not affected. Nonetheless, additional factors should be queried in future studies to validate this hypothesis. In murine ATRT-SHH tumors, *Sox2* is expressed by mid/hindbrain progenitors, which maintain *Sox2* expression in the adult brain as a marker for the neuronal lineage after gastrulation. This could explain why murine and human ATRT-MYC tumors rarely express *Sox2*, whereas ATRT-SHH tumors are strongly positive for *Sox2*. With respect to human MYC tumors, the transcriptome and methylome landscapes of human and murine migratory and gonadal PGCs are broadly similar at comparable stages. However, human PGCs display unique features, e.g., they do not express *SOX2* in contrast to murine PGCs^40^.

Regarding the frequency of tumor formation, we observed an overall low penetrance and latency in the murine tumors derived from heterozygous *Sox2-cre::Smarcb1*^*fl/+*^ (penetrance of 18%) and inducible *Sox2-cre*^*ERT2*^*::Smarcb1*^*fl/fl*^ (penetrance of 22%) mouse models lacking *Smarcb1* expression in comparison to tumors derived from the ubiquitous *Rosa26-cre*^*ERT2*^*::Smarcb1*^*fl/fl*^ (penetrance of 40%). Having the difference in tumor frequency between the ubiquitous and promoter specific models suggests that there might also be other cells of origin, which do not express *Sox2* during tumor initiation and thus cannot be detected by our Sox2-cre models.

Assuming that *Smarcb1* deletion in *Sox2*-expressing PGCs occurs during their migratory phase, mutated PGCs could accumulate dysfunctionalities leading to misguidance. For instance, mismigrated PGCs were found along nerve branches, resulting in human germ cell tumors that also occur at extragonadal sites, mostly along the midline axis of the body, including the brain^24,40^ or in the head and neck region^62,63^. Similar to these tumors, RT of the MYC subgroup occur in close proximity to nerve branches like the trigeminal nerve (also described in human case reports), at nerve cords lateral of the cerebellum, the spine^64–66^ and in the soft tissue of the head and neck region^67^, in human as well as our mouse models. Supporting our hypothesis, we found that the *Dnd1* gene, which is specifically expressed by migratory PGCs and known as a major determinant of cell identity guiding cells toward the genital ridge^41^, is lost in MYC tumor cells. Taken together, we assume that PGCs are one of potentially different COO of RT which are responsible for MYC tumors in the brain and soft tissue, while MYC tumors of the kidney and liver may rather be derived from different COO.

Germ cell tumors are rarely caused by somatic driver mutations, but typically arise through reprogrammed PGCs as one of their known COO^24^. Since their epigenetic reprogramming is dependent on distinct latent potency states based on chromatin remodeling^40^, loss of *Smarcb1* (as core member of the SWI/SNF chromatin remodeling complex^10,68^) as a first hit for RT induction may have major implications for these cells. SWI/SNF complexes without SMARCB1 are functional; however, their stability, recruitment and remodeling activities are altered^59,69^. Indeed, the observed differences between MYC tumor cells and normal fetal PGCs are mainly based on chromatin remodeling and different expression of DNA and histone modifying genes. Our study uncovered that murine MYC tumors exhibit subclones that are distinct from other tumor subpopulations and express epigenetic modifiers like *Dnmt1* or *Ezh2*. In addition, these epigenetically-driven subclones are mitotically active and express genes that are related to aurora signaling, DNA repair and telomere maintenance. These cells might be associated with a more stem-like, dedifferentiated cellular phenotype^70–72^. Due to the fact that PGCs are migratory and proliferating cells in which telomerase is active giving them unlimited replicative capacity, these cells intrinsically harbor hallmarks of cancer^73^. Although there are discrepancies between genuine PGCs and the subset of mitotically active and epigenetically-driven tumor cells, we suggest that this subpopulation is most closely related to their ancestors and susceptible to epigenetic therapeutic interventions. Many epigenetic regulators are expressed in migratory PGCs to ensure active DNA demethylation and genome-wide hypomethylation^37^. Interestingly, when comparing the DNA methylation pattern of RT of the MYC subgroup to PGCs as one of their COO, these tumors are clearly hypermethylated. Further, they only show a low expression of the aforementioned epigenetic regulators which are strongly expressed by PGCs. This, together with the activity of methylating enzymes in MYC RT, might cause the observed hypermethylation of tumor cells compared to PGCs, besides the already shown hypermethylation of SHH tumors compared to neuronal tissue^7^. In conclusion, ATRT MYC and ATRT SHH in comparison to their potential COO have a hypermethylated DNA. This prompted us to target Dnmt expression in RT using DNA-demethylating drugs (DNMT inhibitors) resulting in RT growth arrest in vitro and in vivo in two independent xenograft models. Further, DAC treatment provoked a G1 cell cycle arrest which was previously observed after re-expression of *SMARCB1*^74^. This suggests cell differentiation (upon entering G1 cell cycle phase^75^) and deficiency to maintain the plastic cell state. This is in concordance with differential expression analysis data on RT cell lines before and after *SMARCB1* re-expression^59^, pointing at similar cellular downstream effects upon DAC treatment or *SMARCB1* re-expression. In addition, upregulated genes upon DAC treatment are solely related to cell migration, which is a strongly enriched process in migratory PGCs during the vulnerable time window of tumor induction. Thus, it can be assumed that DNMT inhibition leads to a reversion of the methylome toward the tumor progenitor cell type. Taken together, we show that DAC as DNA-demethylating enzyme provides a target-directed therapeutic option for treatments of RT of the MYC and SHH subgroup (TYR being not analyzed in this study) which is based on the hypermethylated genome of these tumors.

We propose a model, wherein *Smarcb1* loss in PGCs leads to a reversal of germ cell specification and misguidance to various body locations and, ultimately, to tumor formation. Cell-intrinsic factors (genetic and epigenetic), as well as extrinsic niche signals, may contribute to this process.

In the future, it will be important to perform fate-mapping experiments to study the migration route of *Smarcb1*-mutated PGCs and to clarify the reasons for the long latency of tumor formation in mice. As we hypothesize that microenvironmental signals are involved in the final transformation, they could influence the onset of tumor manifestation. In summary, our study provides insights into the COO of RT, underlying epigenetic clonal heterogeneity and mechanisms driving RT tumorigenesis. Finally, we uncovered a promising approach for targeted therapy of RT of the MYC subgroup as well as of the SHH subgroup by blocking hypermethylation by using DNMT inhibition.

## Methods

### Genetically engineered mouse models

All animal procedures were performed according to the guidelines provided by the local regulatory authorities (reference number TVA-84-02.04.2015.A088; TVA-84-02.04.2016.A066; Government of NRW, Germany) (for animal models view Supplementary Table 1).

*Nestin-cre*^76^, *hGFAP-cre*^77^, *Math1-cre*^2^, *Olig1-cre*^78^, *Sox2-cre*^79^, *Sox2-cre*^*ERT2*,^^79–81^,*Rosa26-cre*^*ERT2*,^^82^ and *Smarcb1*^*fl/fl*,^^25^ mice were obtained from the Jackson Laboratory (https://www.jax.org/). *C57BL/6-Dppa3*^*em1(IRES-Cre)Smoc*^ mice were obtained from Shanghai Model organisms (#NM-KI-00040). Mice were maintained on a C57BL/6 background. By crossing the *Smarcb1*^*fl/fl*^ strain with diverse knock-in mouse lines harboring the c*re* or c*re*^*ERT2*^ coding region under the control of different cell specific (*Nestin*, *hGFAP*, *Math1*, *Olig1*, *Sox2*) or ubiquitous (*Rosa26*) promoters, we obtained *Nestin-cre::Smarcb1*^*fl/fl*^, *hGFAP-cre::Smarcb1*^*fl/fl*^, *Math1-cre::Smarcb1*^*fl/fl*^, *Sox2-cre::Smarcb1*^*fl/fl*^, *Olig-cre::Smarcb1*^*fl/fl*^, *Sox2-cre*^*ERT2*^*::Smarcb1*^*fl/fl*^ and *Rosa26-cre*^*ERT2*^*::Smarcb1*^*fl/fl*^ mice. Fate mapping experiments were carried out using *Sox2-cre*^*ERT2*^*::Smarcb1*^*fl/fl*^ mice crossed with a R26-stop-EYFP reporter line.

Genotyping was carried out by PCR analysis using genomic DNA obtained from ear biopsies. Primers used to detect *cre* transgene: Fw: 5′-TCCGGGCTGCCACGACCAA-3′ and Rv: 5′-GGCGCGGCAACACCATTTT-3′. Primers used to detect *Smarcb1*: Fw: 5′-TAGGCACTGGACATAAGGGC-3′ and Rv: 5′-GTAACTGTCAAGAATCAATGG-3′. Primers used to detect *YFP*: Fw: 5′-AAGTTCATCTGCACCACCG-3′, 5′-CTAGGCCACAGAATTGAAAGATCT-3′; and Rv: 5′-TCCTTGAAGATGGTGCG-3′ and 5′-GTAGGTGGAAATTCTAGCATCATCC-3′.

A single dose (50 mg/kg of body weight) of Tamoxifen [Sigma-Aldrich, #T5648; dissolved at 10 mg/ml in sterile ethanol/corn oil (1:10)] was administered intraperitoneally to pregnant *Sox2-cre*^*ERT2*^*::Smarcb1*^*fl/fl*^ and *Rosa26-cre*^*ERT2*^*::Smarcb1*^*fl/fl*^ mice at E6.5 post-coitum (post-coital plug observation was considered as day 0.5). Mice were monitored daily for at least 30 weeks until neurologic symptoms (impaired gait, tilt head, stereotypy) or distress signs (lethargy, weakness) were observed. Mice were sacrificed by cervical dislocation.

### Cell lines

BT16 cells (RRID: CVCL_M156; ATRT-MYC) were provided by Prof. Dr. Martin Hasselblatt (University Hospital Münster, Germany) and G401 cells (RRID: CVCL_0270; extracranial rhabdoid tumor of the kidney) were purchased from ATCC® (#CRL-1441™). Chla02 were purchased from ATCC® (#CRL-3020). 310-FHTC and 311-FHTC cells were purchased from Fred Hutchinson. Chla266 and BT12 were a gift from Dr. Mark Remke (University Hospital Düsseldorf, Düsseldorf). A204 cells were provided by Prof. Dr. Martin Hasselblatt (University Hospital Münster, Münster).

For viability assays: CHLA-02 310-FHTC and 311-FHTC were grown in Dulbecco’s Modified Eagle Medium, Nutrient Mixture F-12 (DMEM/F12) with 1% penicillin/streptomycin (Gibco™, #15140-122), 0.2% B27 supplement (Gibco) and 0.002% epidermal growth factor (EGF) (PeproTech, #315-09) and fibroblast growth factor (bFGF) (PeproTech, #450-33).

CHLA-266, G401, BT16 and A204 cells were cultured in DMEM with 10% fetal bovine serum (FBS) (Gibco™, #10270-106) and 1% penicillin/streptomycin (Gibco™, #15140-122). BT12 cells were grown in Iscove’s Modified Dulbecco’s Media (IMDM) with 20% FBS (Gibco™, #10270-106), 1% insulin transferrin, selenium solution (ITS-G) and penicillin/streptomycin (vol/vol) (Gibco™, #15140-122).

For long-term experiments: BT16 cells were cultured as adherent cell line in DMEM high glucose formulation supplemented with 16% FBS (Gibco™, #10270-106) and 1% penicillin/streptomycin (Gibco™, #15140-122). G401 cells were cultured as adherent cells in DMEM high glucose (Invitrogen, #11965-092) with 10% FBS (PAA Cell Culture Company, #A15-151), 1% L-Glutamine (Gibco™, #25030-018) and 1% penicillin/streptomycin (Gibco™, #15140-122). For experiments, both cell lines were enzymatically dissociated by using 0.05% Trypsin-EDTA (Gibco™, #25300054).

Cell lines were authenticated by STR profiling through the Institute for Forensic Medicine (University of Münster), and regularly tested to be mycoplasma-negative by PCR analysis.

### Xenograft models

Xenograft model experiments were carried out in male and female, 8 to 12 weeks old immune-compromised NOD/SCID mice (Jackson Laboratory, https://www.jax.org/). Animals were anesthetized using Isoflurane (Isoflurane Forene®, Abbvie, #B506) and transplanted into the flank with either G401 cells (1,000,000 cells), in 14 animals or cells of a freshly obtained relapsed patient sample (500,000 single dissociated cells, referred to as relapse model), in 14 animals. The latter was serially transferred (*n* = 3) a priori to ensure successful tumor engraftment. After the detection of 40–80 mm^3^ big tumors, mice were randomly separated into control mice, injected intraperitoneally with vehicle control, and experimental mice, treated with 1.5 mg/kg DAC using the following treatment protocols. Regarding G401-transplanted animals, *n* = 6 mice were treated with four single doses of DAC per week in two cycles with one week of interjacent observation time, and two additional weeks of observation after the end of the second treatment cycle. Eight mice served as controls and underwent the same procedure using a vehicle control. Concerning the relapse model, experimental mice were treated with 1.5 mg/kg DAC (*n* = 7 mice) or a vehicle control (*n* = 7 mice) three times per week over 3 weeks followed by an observation time period of 3 weeks. In both scenarios, tumor growth was monitored twice a week with a manual caliper. Tumor volumes were calculated using the formula: a * (b)^2^/2 (a and b considered as the two planes of a tumor). If tumors reached 3000 mm^3^ or necrosis was observed, experiments were stopped before the end of the observation period. The maximal tumor size approved by the competent authorities was 3000 ± 300 mm^3^, and this volume was not exceeded. Protocols and animal housing were in accordance with all guidelines provided by the local regulatory authorities (reference number TVA-84-02.04.2012.A241 and TVA-84-02.04.2014.A279; Government of NRW, Germany). We have written informed consent from the patient whose freshly obtained relapse sample was used to create a xenograft model. The Ethikkommission der Ärztekammer Westfalen-Lippe (Germany) approved the study protocol for this procedure.

### Inhibitor experiments

All in vitro and in vivo inhibitor experiments were performed using either of the DNMT1 and DNMT3a inhibitors Decitabine (DAC; 5-Aza-2′-deoxycytidine; Sigma-Aldrich, #3656) and 5-Azacytidine (AZA; Sigma-Aldrich, #A2385) using different concentrations as stated in the appropriate sections. For in vitro use, DAC and AZA were dissolved in dimethyl sulfoxide (DMSO, stock solution 100 mM). For in vivo experiments, DAC was diluted in sterile DMSO/PBS.

### Cell viability experiments

Cells were seeded in 96-well plates (4 × 10^3^/50 µl density) and incubated for 24 h to reach exponential growth. Afterward, cells were treated twice with the DAC inhibitor, 24 h and 96 h after seeding. Each time, 25 µl fresh medium were added as well as the according drugs reaching a final concentration of 10 µM and 1 µM, respectively. Each replicate included 4 negative controls (untreated cells) and one background control (pure medium). All experiments were performed in *n* = 4 technical replicates for both examined concentrations. Six days after the first treatment, cell viability was measured via MTT assay using 10 ml MTT reagent (5 mg/ml MTT dissolved in PBS; Merck M2003-1G) and 4 h incubation time. Resulting formazan crystals were dissolved in 100 ml lysis buffer (isopropanol and 0,04 N HCl; purchased from SAV Liquid Production and Fisher Scientific) and the optical density was evaluated spectrophotometrically at 570 nm and at the reference length of 630 nm by a Multiscan Ascent microplate reader (Thermo Electron Corporation).

### Long-term inhibitor treatment

5 × 10^5^ BT16 cells were seeded at the beginning of the experiment and re-plated every 3 days for 14 days in total. Cells were treated every 3 days either with vehicle (DMSO) or 0.5 µM DAC as final concentration. For subsequent cell cycle and apoptosis assays, cells were harvested every 3 days, counted, and further processed as mentioned below. At the end of the experiment, at day 14, cells were harvested and RNA was extracted for microarray profiling.

### Apoptosis assay

For apoptosis assays, cells were incubated with different concentrations of drugs as following: BT16 and G401 tumor cells were treated either with control (DMSO) or with different concentrations of the inhibitors DAC (1 and 50 µM for BT16 cells; or 1 and 10 µM for G401 cells) or AZA (1 and 50 µM) during 6 days. Treatments were applied on the first and the third day after experiment start. At day 6, cells and supernatants were harvested, pelleted at 1200 rpm for 5 min, and supernatants were discarded. Apoptosis assays were conducted using the FITC Annexin V Apoptosis Kit I (BD Pharmingen™, #556547) according to the manufacturer’s protocol. Pelleted cells were incubated with 100 µl of a priori prepared Annexin V-propidium iodide solution (100 µl 1× buffer supplemented with 3 µl of Annexin V solution and propidium iodide solution (BD Biosciences, #556463) for 15 min at room temperature in the dark. Afterward, 900 µl of 1× buffer were added to the cell suspension and apoptotic cell fractions were measured by Flow Cytometry (FACS Canto II flow cytometry 280 system) and quantified by utilizing the software FlowJo 10.1r1 (FlowJo LLC). Experiments were carried out n = 3 in technical replicates. All further calculations and statistical analyses were performed using Graph Pad Prism 7.0 software.

### Cell cycle analysis

Regarding cell cycle assay, 100 μl of cell suspension (harvested as for apoptosis assay) were incubated with 4’,6-diamidino-2-phenylindole (DAPI) solution (AppliChem, #A4099) and measured using FACS Canto II flow cytometry 280 system. Data were analyzed with the software FlowJo 10.1r1 (FlowJo LLC). All further calculations and statistical analyses were performed using Graph Pad Prism 7.0 software.

### Immunohistochemistry

Immunohistochemical (IHC) procedures as well as Hematoxylin and Eosin (H&E) staining were performed as described previously^26^. H&E staining and IHC staining of 3 µm sections of formalin-fixed, paraffin-embedded murine tumor tissues were performed on a Ventana BenchMark XT using the ultraView Universal DAB Detection Kit (Roche, #760-500) following the manufacturer’s instructions. The following antibodies were used: 1:100 anti-KI67 (Abcam, #ab15580), 1:50 anti-SMARCB1 (Clone 25/BAF47, BD Bioscience, #612110), 1:100 anti-Hepar (Roche, #1027846), 1:100 Inhibin (alpha Roche, #760-6081), 1:50 anti-CD3 (Abcam, #ab16669), 1:100 anti-CD45 (Abcam, #ab10558), 1:200 anti-Sox2 (Abcam, #ab97959).

Regarding fate mapping experiments, eight to 12 weeks old (males and females) E6.5 induced *Sox2-cre*^*ERT2*^*::Smarcb1*^*fl/fl*^ mouse brains carrying a recombined heterozygous EYFP reporter (R26-stop-EYFP^*fl/+*^) were fixed in 4% paraformaldehyde overnight, immersed in 30% sucrose for 2 days, followed by tissue embedding in Tissue-Tek® O.C.T.™ Compound (Sakura Finetek™, #4583). Brain tissues were sectioned serially (sagittal) into 10 µm thick slides. After rehydration, sections were permeabilized with 1% Triton X-100 for 10 min, washed 3 times in PBS supplemented with 0.1% Tween and stained with DAPI (Sigma Aldrich, #D9542) solution (1 µg/ml) for 10 min. After 3 washing steps, sections were embedded using Mowiol solution (Roth, #4-88), imaged using the Vectra® 3.0 imaging system and viewed using the image analysis software Phenochart™ and InForm®.

### Murine tumor sample processing and cryopreservation

Murine tissue was resected, frozen on dry ice and preserved at −80 °C. Tissues were embedded in Tissue-Tek® O.C.T.™ Compound (Sakura Finetek™, #4583) and serially sectioned (5–8 μm) using a cryostat.

### RNA isolation and bulk sequencing of murine tumors and human cell line

Serial-cryosections (described above) of *Sox2-cre* (constitutive *Sox2-cre::Smarcb1*^*fl/+*^ and inducible *Sox2-cre*^*ERT2*^*::Smarcb1*^*fl/fl*^) and inducible *Rosa26-cre*^*ERT2*^*::Smarcb1*^*fl/fl*^ derived tumors were stained with H&E to detect tumor areas. Tumors were dissected from unstained cryosections with a sterile scalpel and total RNA was isolated using the RNeasy Micro Kit (QIAGEN, #74004) according to the manufacturer’s protocol. RNA quality, purity and concentrations were obtained using Bioanalyzer (Software Agilent 2100; Agilent Technologies, Inc). Murine tumor samples were profiled on Affymetrix GeneChip™ Mouse Gene 2.0 ST Array at the Genomics Core Facility of Regensburg (Kompetenzzentrum Fluoreszente Bioanalytik, Regensburg University) (for sample summary view Supplementary Table 2).

### Murine tumor sample processing and single-cell RNA-sequencing

For single-cell preparation, fresh murine tumors (for sample summary view Supplementary Table 3) were minced using scalpels prior to enzymatic dissociation with StemPro™ Accutase™ (Gibco™, #A1110501) for 20 min at 37 °C. Treatment was accompanied by mechanical dissociation and stopped by PBS dilution and washing steps. Erythrocytes were lysed using ACK lysing buffer (Gibco™, #A1049201) according to the manufacturer’s protocol. Fluorescence-activated cell sorting (BD FACS Aria II) was performed to remove non-viable, 7-AAD-positive cells (eBioscience™, #00-6993-50) prior to manual counting of sorted cells using Trypan blue staining.

Single cell suspension was processed for single-cell RNA-sequencing (scRNA-seq) using Chromium Single Cell 3’ Gel Bead Kit v2 (10X Genomics) according to the manufacturer’s protocol. In short, single-cell GEMs (Gel Beads in Emulsion) were generated on the Chromium Controller, followed by GEM-RT, Dyna Beads cleanup, cDNA amplification and SPRIselect beads cleanup. The Library Bead Kit and Chromium i7 Multiplex Kit was used for generating indexed single-cell libraries for Illumina sequencing. Quality, purity, size and concentrations of cDNA and libraries were determined by Tapestation 2000 (Agilent Technologies, Inc). Libraries were sequenced using the Next-Seq 500 sequencing platform (high-output Kit, 75 Cycles v2 Chemie) at the Genomics Core Facility (University Hospital Münster, Münster).

### Microarray profiling of treated human cell line

Regarding DAC-treated and control BT16 cells which were harvested at day 14 of the long-term treatment experiment (mentioned above), RNA was isolated using RNeasy Mini Kit (QIAGEN, #74104) from cell pellets according to manufacturer’s protocol. Tumor cells were profiled on Affymetrix U133 plus 2.0 human Array at the Genomics and Proteomics Core Facility of the German Cancer Research Center (DKFZ, Heidelberg).

### Gene expression analysis: mouse and human data

Murine tumor samples from three different runs were profiled on Affymetrix GeneChip Mouse Gene 2.0 ST Array (first run: *n* = 9; second run: *n* = 16; third run: *n* = 16). Human gene expression data were retrieved from GEO database, considering the following sources: (i) GSE70678: 49 human ATRT samples [Affymetrix Human Genome U133 plus 2]^7^, (ii) GSE28026: 18 human ATRT samples [Affymetrix Human Genome U133 plus 2]^83^. Integration of gene expression measures from multiple platforms was performed as described in Pöschl et al.^84^. In particular, data from each batch on each platform were normalized separately (using RMA), merged in two distinct datasets (by species) and known batch effects were removed by ComBat (sva package). In order to ensure that in a platform each gene was only represented once, annotation of probe-sets was performed, and multiple probes merged by median. We included only orthologous genes retrieved with package biomaRt^85^. This resulted in a total of 15,004 genes. Relative gene expression values were calculated subtracting from each gene expression value the mean value across all samples and dividing the result by the standard deviation across all samples. Exploratory analyses were performed using PCA, UMAP^28^ and hierarchical clustering. A smaller number of genes was considered (*n* = 5000), selecting only those genes whose expression values showed maximal variation across the samples being analyzed (using MAD, median absolute deviation). Moreover, we considered Euclidean distance as measure of similarity between samples, and we selected Ward’s method for clustering (function hclust, method “Ward.D2”). The R package limma^86^ was used for differential expression analysis, considering the adjusted *p*-value (adj.P.val) with Benjamini & Hochberg method (threshold = 0.01), and the logarithm of the fold change (logFC) (threshold = ±2). Once the list of differentially expressed genes was obtained, we conducted a functional annotation using ToppGene Suite^87^. In particular, we considered GO terms enriched with an adjusted *p*-value (FDR) < 0.05. All analyses were performed in R (version 3.4.2) and Bioconductor (version 3.6).

### Single-cell RNA-sequencing of mouse tumors

Single-cell RNA-seq raw data of 13 samples (ATRT-SHH: *n* = 2; ATRT-MYC: *n* = 5; eRT: *n* = 3; MYC-spinal: *n* = 3) was processed by the 10X Genomics software Cell Ranger (version 2.0.2) and the reads aligned to the mouse reference genome (mm10, v1.2.0). Default parameters were used to retain valid barcodes and unique molecular identifiers (UMIs). Quantitative information for each sample is reported in Supplementary Table 3.

### Quality control, preprocessing and normalization

Analysis of scRNA-seq data was performed in R (version 3.6.1), using the package Seurat (version 3.1.3)^32^. All the datasets were loaded in R separately, retaining genes expressed in at least 3 cells, and cells expressing at least 50 genes. After assessing the distributions of number of genes and percentage of mitochondrial genes (percent.mito) by number of UMI counts per sample, we determined filtering cutoffs equal for all samples. In particular, we filtered out cells having a number of genes < 200 and percent.mito > 25%. We chose these permissive thresholds in order not to exclude tumor cells which may have a higher expression of mitochondrial genes for biological reasons, rather than being apoptotic cells. We performed normalization using the Seurat function *NormalizeData* (method = “LogNormalize”, scale.factor = 10,000). Finally, highly variable genes (*n* = 2000) were calculated with the selection method “vst”.

### Integration of multiple samples by subtype

The following analyses were performed separately for each different RT subtype. Mouse samples belonging to each subtype were merged by using Seurat v3 integration function. This integration is based on the concept of “anchor” cells, i.e., cells that share a similar state and are found in the datasets to be integrated. Thus, the algorithm first finds “anchor” cells between each pair of samples and then uses these anchors to harmonize the datasets. Each sample was considered as one batch. This procedure is meant to correct for possible batch-effects present in the data, while maintaining biological variation. After this step, we performed scaling, PCA, dimensionality reduction (using UMAPs) and clustering on the integrated dataset (see Supplementary Table 4). Differential expression analysis was computed on the unintegrated data using MAST algorithm^88^. Statistically significant genes were considered having a q-value < 0.05 (Bonferroni correction).

### Single-cell RNA-sequencing of mouse embryos

Early stages mouse embryo preprocessed data^30^ were downloaded following the instructions at https://github.com/MarioniLab/EmbryoTimecourse2018. This dataset contained a total of ~139,000 cell transcriptomes over stages of embryonic development ranging from E6.5 to E8.5, generated by the 10X platform. Following the original manuscript, we excluded poor-quality cells and considered the authors’ cell type annotation, provided with the data. Similar considerations were made for the later stages mouse embryo dataset^31^. Data from preprocessed, filtered, high quality cells was downloaded from https://oncoscape.v3.sttrcancer.org/atlas.gs.washington.edu.mouse.rna/downloads, alongside the cell metadata. In this case, cell transcriptomes were generated by a different protocol, namely sci-RNA-seq3; in total, we obtained ~1 million cells, spanning stages from E9.5 to E13.5. In order to reduce computational time, we randomly sampled 50,000 cells from each of the two embryo datasets, making sure that the relative proportions of the different cell types were maintained before and after subsampling. Data integration was performed using the same Seurat integration algorithm as in mouse tumors, treating each dataset as one batch. Integrated data were then used for dimensionality reduction as previously described. Moreover, we performed a cell label harmonization between the two datasets, resulting in a total of 69 different cell types. Even though the two datasets were not overlapping from a developmental point of view, the data integration was able to correctly group together similar cell types, following the differentiation line (e.g., erythrocyte clusters, see Supplementary Fig. 7A).

### Quantification of cell similarity between embryos and tumors

To get insights into the putative cell of origin of RT, we followed a computational method proposed by Young et al.^33^ that is based on logistic regression. Taking the integrated embryo dataset as reference atlas, we trained a logistic regression model that could predict the similarity between each embryo cell type and the tumor cell clusters, individually for each tumor subtype. The logistic regression model was implemented in R using the package glmnet^89^. In particular, we used the embryo cell types (unintegrated counts) as training data, applying elastic net regularization (alpha = 0.8) to prevent the exclusion of strongly co-linear genes. One cell type (lens) was excluded, as composed by only three cells. Moreover, the model was trained on a subset of genes (16,267), since we decided to exclude potentially biasing genes associated to the GO terms mitochondrion (GO:0005739), ribosome (GO:0005840) and cell cycle (GO:0007049).

A series of N *one-versus-rest* binomial logistic regression models was trained, with N being the number of cell types in the training data, to obtain regression coefficients specific to each cell type. As in Young et al.^33^, we used an offset for each model equal to ${\log }(\frac{f}{{1}-{f}})$, with *f* being the fraction of cells belonging to the cell type trained. This was done to prevent the observed frequencies of cells from biasing the regression coefficients. For each binomial regression, 10-fold cross validation was applied.

Finally, we predicted the similarity of each embryo cell type with RT cells, individually for each tumor subtype. During prediction, we used an offset of zero. Probabilities were then averaged over all tumor cells to obtain a final similarity score. As internal negative controls, we used cell types that are biologically unlikely to represent the cell of origin of RT (e.g., erythroid, primitive erythroid lineage, hepatocytes), and checked that the similarity scores for these cell types were correctly close to zero. Moreover, we tested the significance of the scores predicted by the logistic regression using a permutation test (one-sided). In particular, we randomly permuted (1000 times) the cell type labels and calculated the average score across all cells, per celltype. This generated a null distribution of our test statistics (average score per celltype). Then, we calculated the proportions of means that were as or higher than the actual mean and considered this as the exact p-value. In MYC RT this calculation resulted in a *p*-value of 0.024 for PGCs in comparison to other potential cells of origin (Supplementary Data 1).

### Single-cell RNA-sequencing of mouse PGCs

We obtained access to scRNA-seq data of mouse PGCs from Mayère et al.^35^. The data, processed with 10X, contained 14,750 high-quality mouse primordial germ cells, covering five embryonic developmental stages between E10.5 and E16.5. We combined these cells with the early stages PGCs from Pijuan-Sala^30^ (356 cells from E6.75 to E8.5) and performed trajectory analysis using Monocle 3^31^. Given the very low number of annotated PGCs in the embryo atlas from Cao et al.^31^ (165 cells from E9.5–E13.5), we decided not to consider this dataset for further analyses. Trajectory inference was computed over the aligned dataset, integrated with the default algorithm MNN^90^, using UMAPs as dimensionality reduction. Moreover, we performed differential expression analysis between PGCs and tumor subtypes, using the Seurat uncorrected data and MAST algorithm, as previously described. Cell cycle phases were calculated for both PGCs and tumor cells using a 98-genes list from Tirosh et al.^48^ and the Seurat function *CellCycleScoring*.

### Bulk RNA-sequencing of human RT cell lines

Bulk RNA-sequencing data of 16 samples were obtained from Wang et al.^59^ study (GEO:GSE71505). In particular, we downloaded raw fastq files from SRA (accession: SRP061772) corresponding to BT16 cell line. After explorative quality control with FastQC^91^ and MultiQC^92^, we used Salmon^93^ for pseudo-alignment and quantification of the samples to the human transcriptome (downloaded from Ensembl, release 94). Default parameters were used. Further analyses were performed in R. We employed the Bioconductor package tximport^94^ to summarize transcript-level estimates computed by Salmon for a gene-level analysis. To find differentially expressed genes, we used the package DESeq2^95^ and tested for SMARCB1 re-expression *versus* control conditions. Only genes with adjusted p-value < 0.05 were considered (Benjamini–Hochberg correction).

### Analysis of human ATRT patient data

Gene expression array data of human ATRT patients was retrieved from the European Genome-phenome Archive (EGA) under accession numbers EGAD00010000789, EGAD00010000790, and EGAD00010001546^34^. We considered the normalized values computed by the original authors for the first two batches, while we processed the raw data of the third batch with the beadarray R package^96^. A total number of 89 samples were considered for the analysis. Raw expression was log-normalized with the quantile method, to ensure inter-sample comparability. Features were annotated using the IlluminaV4 package and we summarized duplicated probes by their mean value into gene symbols. Finally, the function ComBat (sva package) was employed to remove batch effects and combine the three datasets together. Subgroup classification was taken from the Supplementary Tables of Torchia et al.^34^ (subgroup 1 = SHH, subgroup 2 = TYR, subgroup 3 = MYC). Gene signatures corresponding to either PGC or mid-hindbrain markers were used to perform an unsupervised hierarchical clustering using the R package pheatmap, with euclidean distance metric and ward.D2 clustering algorithm.

### DNA methylation analysis for human fetal PGCs and MYC-RT

Whole-genome bisulfite sequencing (WGBS) data for human PGCs was retrieved from GEO, with accession number GSE63818^22^. For human RTs, we used methylation profiles (Illumina HumanMethylation450 BeadChip (450k) array) from the published dataset accessible in GEO:GSE70460^7^. Given the different platforms of the two datasets, we used an adapted version of methyLiftover^97^ to convert the WGBS data to a version compatible with 450k arrays. Global methylation levels were computed for PGCs (*n* = 13, ranging week7-week19) and MYC-RT (*n* = 33) by averaging beta values across each phenotype.

### Bulk RNA-sequencing of rhabdoid tumors generated in *Dppa3-cre::Smarcb1*^*Fl/+*^ mice

RNA was isolated from tumor tissue using RNeasy Mini Kit (QIAGEN, #74104) according to the manufacturer’s protocol. RNA quality, purity and concentrations were measured using Bioanalyzer (Software Agilent 2100; Agilent Technologies, Inc). RNA sequencing was carried out by the Core Facility of the University Hospital Münster, Germany. For this, the ultra II RNA directional library prep kit for Illumina (New England Biolabs, #E7760S) was used and samples were sequenced using the Next-Seq 2000 sequencing System (1 × 72 Cycles, 25 Mio single reads/sample) at the Genomics Core Facility (University Hospital Münster, Münster). Raw FASTQ files of the two *Dppa3-cre::Smarcb1*^*Fl/+*^ mice were aligned to the mouse reference genome using STAR (version 2.7.9a). The mouse reference Fasta files and GTF annotation used to build the genome index were downloaded from Ensembl (version 104). Default parameters were used. Next, we used the R function *featureCounts* (from the package Rsubread, v2.0.1) to count mapped reads and generate gene expression matrices. We compared these two samples to a published dataset of murine ATRT tumors that was retrieved from GEO (GSE137633), for which the tumor subgroup was known from the original authors. We downloaded pre-processed gene expression matrices and merged these samples (3 ATRT-SHH, 4 ATRT-MYC and 4 extracranial tumors) with our 2 Dppa3-tumor samples. We applied the “variance stabilizing transformation” implemented by the R package DESEQ2 to normalize the expression of the two datasets and calculated an unsupervised hierarchical clustering on the inter-sample distances computed over a list of known subgroup-specific genes (from Johann et al.^7^).

### Reporting summary

Further information on research design is available in the Nature Research Reporting Summary linked to this article.

## Supplementary information

Supplementary Information

Reporting Summary

## Data Availability

Single-cell RNA-seq data generated in this study have been deposited in NCBI’s Gene Expression Omnibus and are accessible through GEO Series accession numbers GSE141532 (for ATRT-SHH and ATRT-MYC samples) and GSE188815 (for eRT and MYC spinal samples). Gene expression array data of the murine models generated in this study are available through GEO Series accession number GSE188654. Two human ATRT datasets of gene expression data (Birks et al.^83^; Johann et al.^7^) were retrieved in GEO under accession numbers GSE70678 and GSE28026. Early stages mouse embryo data were retrieved from https://github.com/MarioniLab/EmbryoTimecourse2018 and https://oncoscape.v3.sttrcancer.org/atlas.gs.washington.edu.mouse.rna/downloads. Mouse embryonic germ cells data from Mayère et al.^35^ is available in GEO under accession number GSE136220. Bulk RNA-sequencing data of human rhabdoid tumor cell lines^59^ is deposited under GSE71505. Illumina array data of human ATRT samples from Torchia et al.^34^ can be found in EGA at EGAD00010000789, EGAD00010000790 and EGAD00010001546. Whole-genome bisulfite sequencing (WGBS) data for human PGCs was retrieved from GEO, with accession number GSE63818^22^. For human RTs, we used methylation profiles (Illumina HumanMethylation450 BeadChip (450k) array) from the published dataset accessible in GEO:GSE70460^7^. Bulk RNA-seq data of *Dppa3-cre::Smarcb1*^*Fl/+*^ mice generated in this study have been deposited in GEO with accession number GSE188816. A published dataset of bulk RNA-seq of murine ATRT tumors was retrieved from GEO (GSE137633). Source data are provided with this paper.

## Source data

Source Data file

## Code availability

Code used to analyze the datasets is publicly available at https://github.com/martaint/AGKerl_RTcoo_analysis_scripts. The source code is also citable, as deposited in Zenodo^98^.
