# Supplementary material for: Single-cell transcriptomics identifies potential cells of origin of MYC rhabdoid tumors
Source: Nat Commun. 2022 Mar 22;13:1544. doi: 10.1038/s41467-022-29152-4 (PMC8941154; doi:10.1038/s41467-022-29152-4)
Supplement: Supplementary file 1 — Supplementary Information [file 41467_2022_29152_MOESM1_ESM.pdf]

Supplementary Information

Primordial germ cells identified as one potential cell of origin of MYC rhabdoid tumors

Monika Graf[1*], Marta Interlandi[1,2*], Natalia Moreno[1*], Dörthe Holdhof[3,4], Carolin Göbel[3,4], Viktoria Melcher[1], Julius Mertins[5,6], Thomas K. Albert[1], Dennis Kastrati[1,7], Amelie Alfert[1], Till Holsten[3,6], Flavia de Faria[1,8], Michael Meisterernst[6], Claudia Rossig[1], Monika Warmuth-Metz[9], Johannes Nowak[9,10], Gerd Meyer zu Hörste[11], Chloe Mayère[12,13], Serge Nef[12,13], Pascal Johann[14,15], Michael C. Frühwald[14], Martin Dugas[2,16], Ulrich Schüller[3,4,17†], Kornelius Kerl[1†#]

*These authors contributed equally

[†] These authors jointly supervised this work

Supplementary Figures 1-12

Supplementary Tables 1-5

Supplementary Figures

Supplementary Figure 1

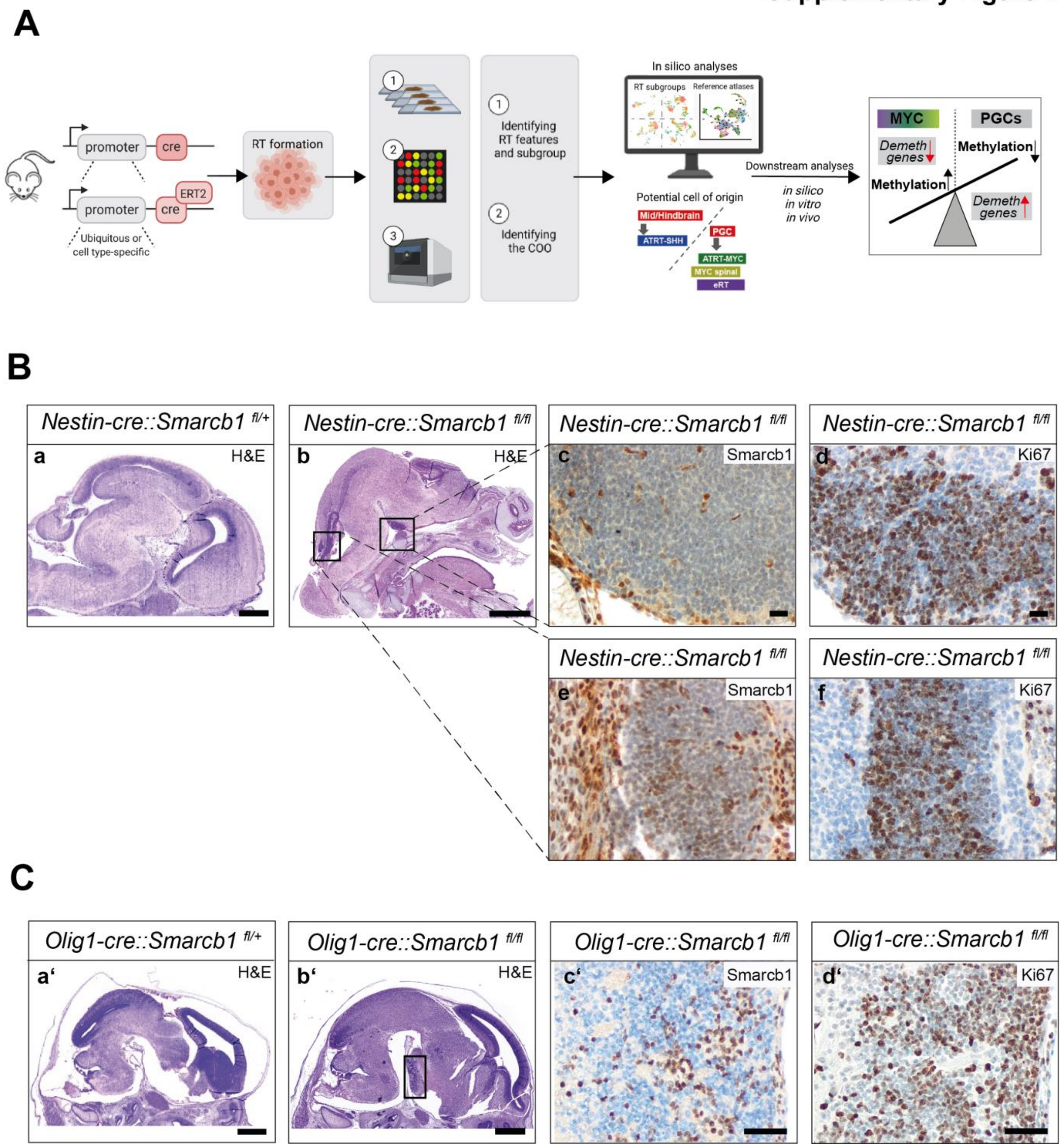

Supplementary Figure 1. Project workflow and histological characterization of lethal homozygous *Smarcb1*-deficiency in early embryonic Nestin- or Olig1-positive cells. A, Graphical representation showing the overall design of the study. Briefly, after generating different mouse models, we obtained rhabdoid tumors that were characterized by using: 1) histology, 2) bulk RNA sequencing and 3) scRNA-seq. Based on the merged analysis of our murine bulk samples and published human ATRT samples[7,85], we could assign the murine tumors to the ATRT subgroups, SHH or MYC. Next,

applying computational similarity analyses on single-cell transcriptomes of murine RT cells and embryonic reference cell types, we sought to uncover the cellular origins of RT. Further, *in silico*, *in vitro* and *in vivo* analyses revealed a deregulated transcriptome and epigenome of RT of the MYC subgroup compared to the identified potential COO. Created with BioRender.com. **B and C,** Representative images of H&E-stained E14.5 fetal brain sections (sagittal). (a) *Nestin-cre::Smarcb1*$^{fl/+}$ (n=4) and (a') *Olig1-cre::Smarcb1*$^{fl/+}$ (n=5) control embryos showed normal brain development, while (b) *Nestin-cre::Smarcb1*$^{fl/fl}$ (n=6) mice and (b') *Olig1-cre::Smarcb1*$^{fl/fl}$ (n=6) mice harbored a loss of Smarcb1*,* as shown in (c, d) and (c'), respectively. Moreover, they presented hyperproliferative regions shown in boxed areas (b and b´) and highlighted in d, f and d´, where strong Ki67 positivity was observed . Scale bar = a, b, a', b'=1 mm; c, d= 200 µm; c', d'= 50 µm.

Supplementary Figure 2

A

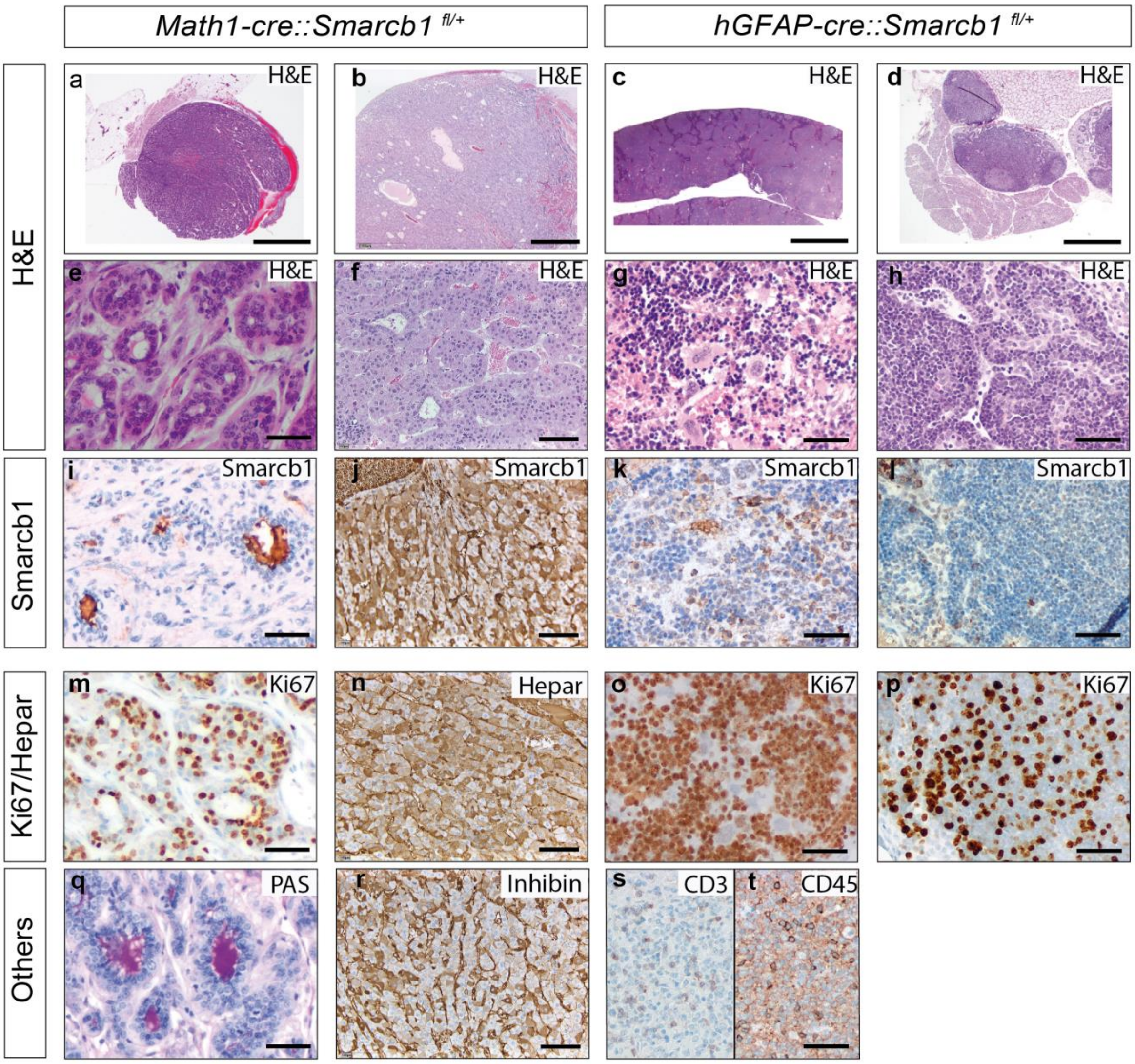

B

	Tumor/localization	Age [weeks]
hGFAP-cre::Smarcb1 fl/+	adenocarcinoma/ salivary gland	5
	adenoma/hipophysis	77
	adenocarcinoma / parotid gland	60
	lymphoma/spleen	105
	adenocarcinoma/ subcutaneous thorax	83
	lymphoma/spleen	102
Math1-cre::Smarcb1 fl/+	hepatocellular cacinoma/ liver	62
	intestinal tumor/intestine	72
	adenocarcinoma /parotid gland	72

Supplementary Figure 2. Loss of heterozygosity in *Smarcb1* gene leads to formation of different tumor entities except RT. A, Representative H&E staining (two upper rows) of tumor samples derived

from constitutive *hGFAP-cre::Smarcb1*$^{fl/+}$ (n=6) and *Math1-cre::Smarcb1*$^{fl/+}$ (n=3) mice. Tumors were detected in different locations such as subcutaneous mass in the thorax (first column, a to q), in the liver (second column, b to r), in the spleen (third column, c to t) and in the parotid gland (fourth column, d to p). All tumors were characterized by loss of Smarcb1 (i, j, k, l). Staining for proliferation marker *Ki67* was positive in most of the tumors (m, o, p). Specific stainings were performed to better characterize the tumor entities. For example PAS for adenocarcinoma (q), Hepar (n) and Inhibin (r) for hepatocellular carcinoma, Cd3 (s) and Cd45 (t) for lymphoma. Scale bar indicates 1 mm (b), 200 µm (a, c, d), 100 µm (f, j, n, r), 50 µm (in all remaining pictures). **B,** Summary of non-rhabdoid tumor cases presented in *hGFAP-cre* and *Math1-cre* mice, all harboring heterozygous loss of *Smarcb1*, and their most probable diagnosis based on stainings, localization, morphology and pathologist's experience.

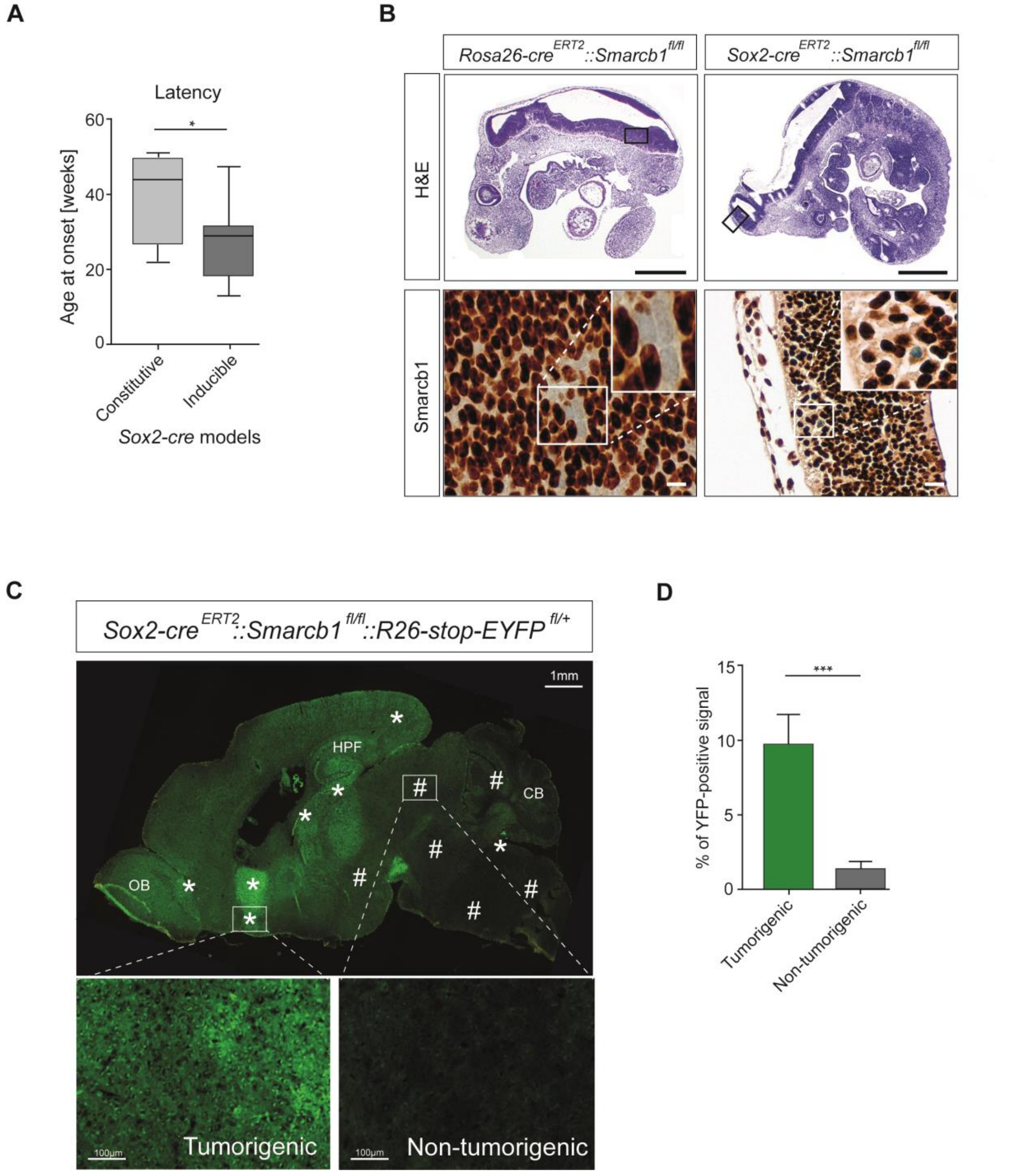

Supplementary Figure 3. Investigations on the latency, tumor penetrance and fate mapping in GEMMs developing RT. A, Boxplots depicting a significant difference in the age of tumor onset between the constitutive (n=8) and inducible (n=9) *Sox2-cre Smarcb1* knockout models (unpaired t-test, two-tailed, *p = 0.0227). For each box, the lower and upper bounds represent the 25th and 75th percentiles; the center corresponds to the 50th percentile (median). The whiskers go down to the

smallest value and up to the largest. **B,** Representative H&E staining of E10.5 isolated embryos (left end of the figures: anterior; right end of the figures: posterior) of the inducible *Rosa26-cre* (upper left, n=3 stained embryos, representative images are shown) and *Sox2-cre* (upper right, n= 4 embryos, representative images are shown) model systems after the induction of *Smarcb1* loss at E6.5 by a single dose of tamoxifen *in utero*. Anti-Smarcb1 staining shows loss of Smarcb1 protein occurring only occasionally (lower panels). Boxed areas are magnified in insets. Scale bar indicates 1 mm in H&E staining and 20 µm in Smarcb1 protein staining. **C,** Immunofluorescence images of endogenous EYFP signal of a sagittal brain section from an adult (8 weeks old) cell tracing reporter model (*Sox2-cre^{ERT2}::Smarcb1$^{fl/fl}$* crossed with *R26-stop-EYFP* mice; injected at E6.5) to genetically mark *Sox2*-positive cells upon recombination. Boxed areas are magnified and are representative for tumorigenic (left) and non-tumorigenic (right) brain areas in the underlined mouse model. Scale bar = 1 mm for the overview image and 100 µm for the 20x magnification. As help for brain orientation: OB, olfactory bulb; HPF, hippocampal formation; CB, cerebellum. **D,** Bar graph depicting the proportion of EYFP-positive signal detected in tumorigenic *versus* non-tumorigenic areas of the *Sox2-cre^{ERT2}::Smarcb1$^{fl/fl}$*::*R26-stop-EYFP$^{fl/+}$* mouse model. Quantification was done using five to eight sections from two biological replicates (brains derived from eight to ten weeks old mice) (SEM is shown; unpaired t-test, two-tailed ***p = 0.0003). Source data are provided as a Source Data file.

Supplementary Figure 4

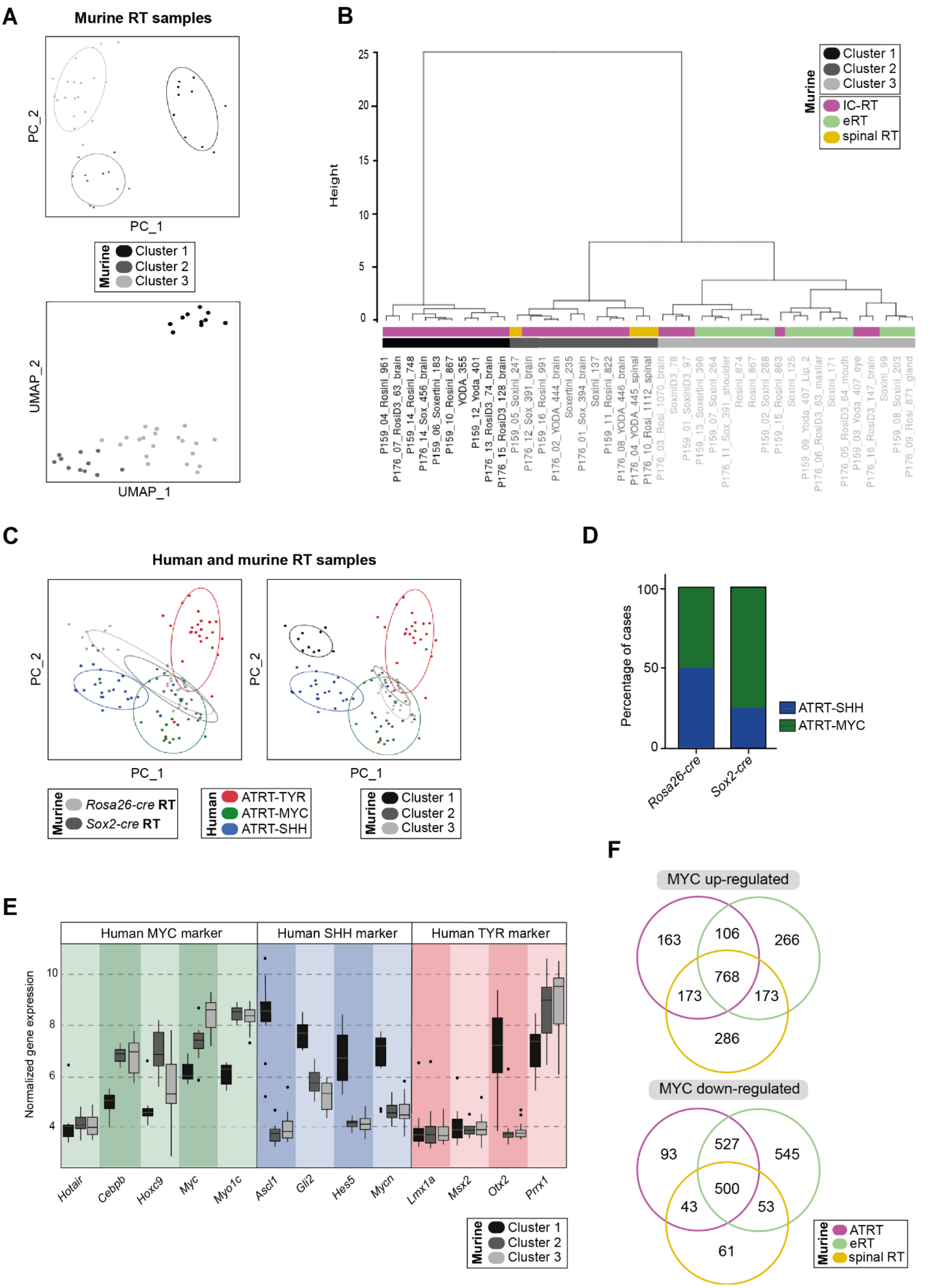

Supplementary Figure 4. Joint analysis of murine and human RT uncovers similarities and assigns molecular subgroups to murine tumors. A, PCA and UMAP plots representing 41 murine

RT samples. Three clusters were found by hierarchical clustering. **B,** Dendrogram of 41 murine RT samples showing localization (IC-RT, eRT, spinal RT) as well as clusters (as in subpanel **A**). **C,** PCA plots of 41 murine RT samples and 67 human ATRT samples. Human samples are colored by the original subgroup annotation (SHH, TYR and MYC). Murine samples are colored by knockout models (on the left) and by clusters (on the right). **D,** Bar graph depicting the percentage of murine ATRT tumors derived from *Sox2-cre* (n=8) and *Rosa26-cre* (n=16) *Smarcb1* knockout models clustering to either ATRT-SHH or ATRT-MYC subgroups. **E,** Boxplots displaying the expression of known gene markers of each human ATRT subgroup, in the three murine clusters (n=41 biologically independent samples, grouped into cluster1: n=10 samples; cluster2: n=11 samples and cluster3: n= 20 samples). For each box, the lower and upper bounds represent the 25th and 75th percentiles; the center corresponds to the 50th percentile (median). The upper whisker extends to the largest value no further than 1.5 * IQR from the bound (where IQR is the interquartile range, or distance between the 25th and 75th percentiles). The lower whisker extends to the smallest value at most 1.5 * IQR of the bound. Data beyond the end of the whiskers are called outlying points and are plotted individually. **F,** Venn diagrams showing the number of intersecting differentially expressed genes in the three murine MYC groups (ATRT, eRT and spinal-RT). Differential expression analysis was performed comparing each of the three MYC groups *versus* murine ATRT-SHH samples. Only genes having |logFC| >= 1 and adjusted p-value <= 0.05 were considered. Source data are provided as a Source Data file.

Supplementary Figure 5

A

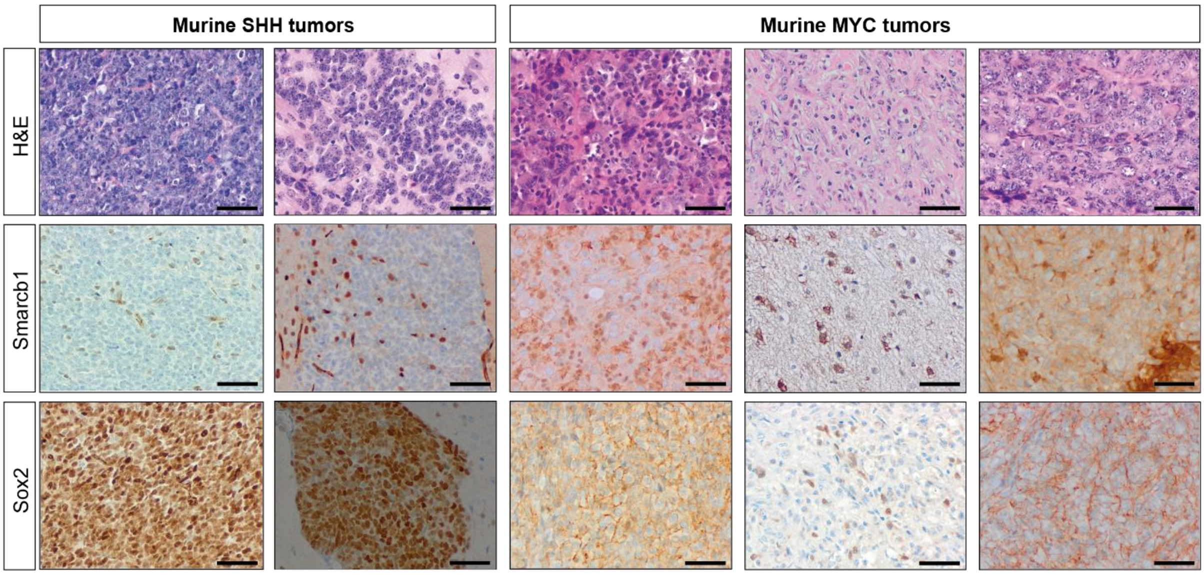

B

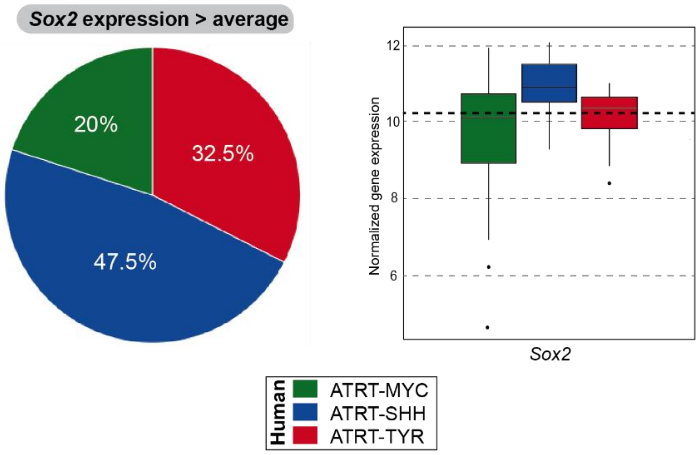

Supplementary Figure 5. ***Sox2*** **expression levels vary between tumor subgroups of both murine and human samples. A,** Representative sections of 5 murine tumors (representative samples selected from the n= 41 murine cohort) belonging to the SHH (left panel) or MYC (right panel) subgroups. H&E, Smarcb1 and Sox2 staining of every tumor is shown. SHH tumors show higher Sox2 positivity than MYC tumors. All tumors are negative for Smarcb1 stain. Scale bar indicates 50 µm. **B,** Pie chart representing the proportion of human tumors that have higher than average expression of *Sox2.* The average value was calculated across all three human subgroups and it is visible on the boxplot on the

right hand side. Here, the expression of Sox2 is plotted by subgroups, with the average value represented as a dashed line (n=67 biologically independent samples grouped in ATRT-TYR: n=22; ATRT-MYC: n=23; ATRT-SHH: n=22). For each box, the lower and upper bounds represent the 25th and 75th percentiles; the center corresponds to the 50th percentile (median). The upper whisker extends to the largest value no further than 1.5 * IQR from the bound (where IQR is the interquartile range, or distance between the 25th and 75th percentiles). The lower whisker extends to the smallest value at most 1.5 * IQR of the bound. Data beyond the end of the whiskers are called outlying points and are plotted individually. Source data are provided as a Source Data file.

Supplementary Figure 6

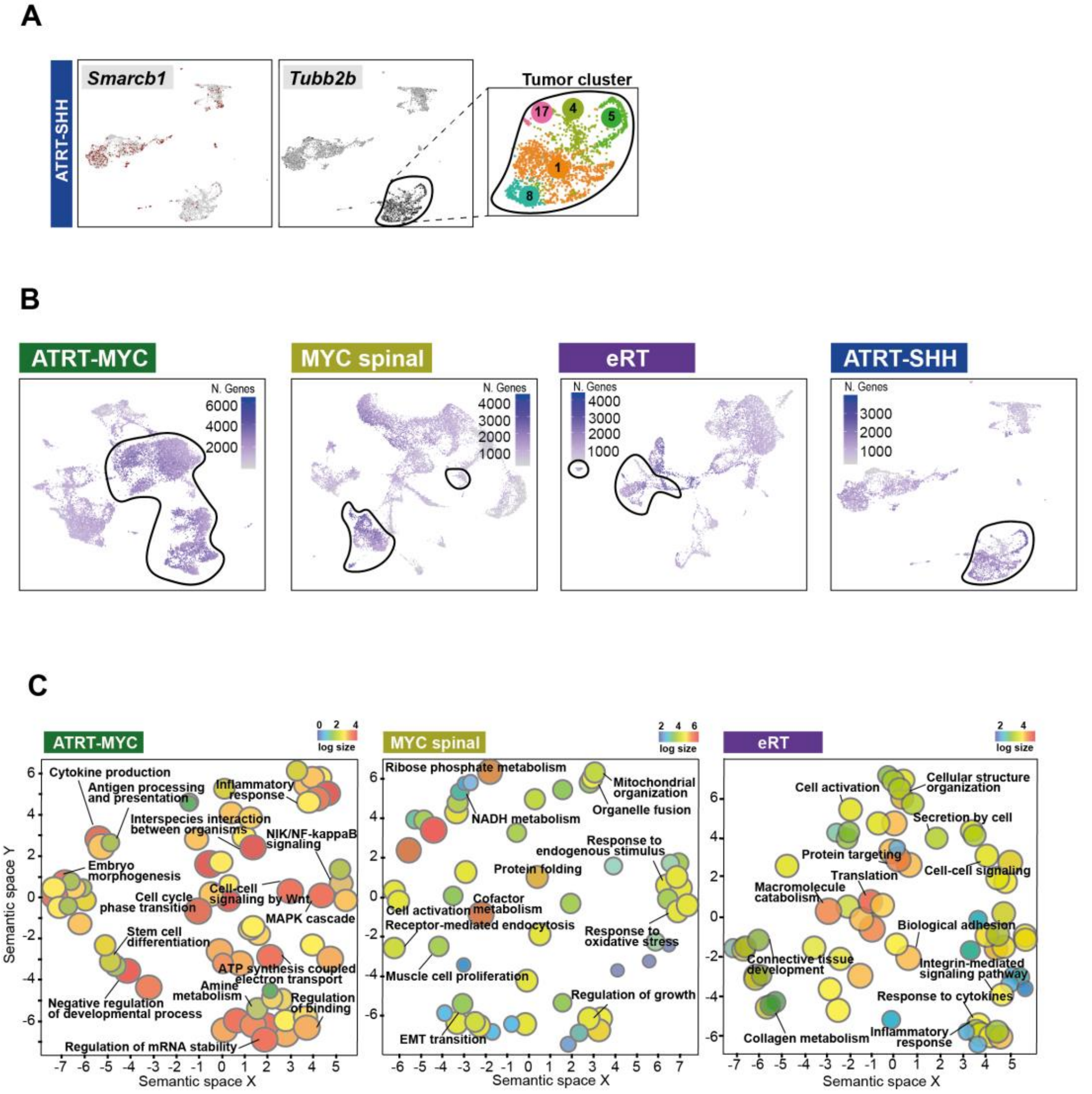

Supplementary Figure 6. RT of the MYC subgroup show only minor intertumoral heterogeneity. A, UMAP plot of murine single cell transcriptomes of the ATRT-SHH subgroup (n= 2 tumor samples). *Smarcb1* negativity (left UMAP) and SHH subgroup-specific *Tubb2b* gene expression (right UMAP) indicate tumor clusters. Circled clusters are magnified in inset and represent tumor clusters. **B,** UMAP plots representing the total number of expressed genes per cell, in the four RT subtypes. Tumor clusters are highlighted in insets. **C,** Overrepresented functional categories of upregulated genes unique for tumor cells of the indicated MYC subtype. Unique genes are derived from the direct comparison between tumor clusters and tumor-associated cells of each subtype, followed by the examination of

overlapping and uniquely expressed genes across all subtypes. Unique DEGs were analyzed using ToppGene and functional categories are clustered and visualized based on similarity using REVIGO in a 2D annotation space (semantic space). Size and color of circles represent the *p* values (-log10 scale) derived from ToppGene. Differential expression analysis used MAST algorithm[90]. Statistically significant genes were considered having a q-value <0.05 (Bonferroni correction). Functional annotation was performed using ToppGene Suite[89] (with statistical method as probability density function) considering GO terms enriched with an adjusted *p*-value (FDR) <0.05. Source data are provided as a Source Data file.

Supplementary Figure 7

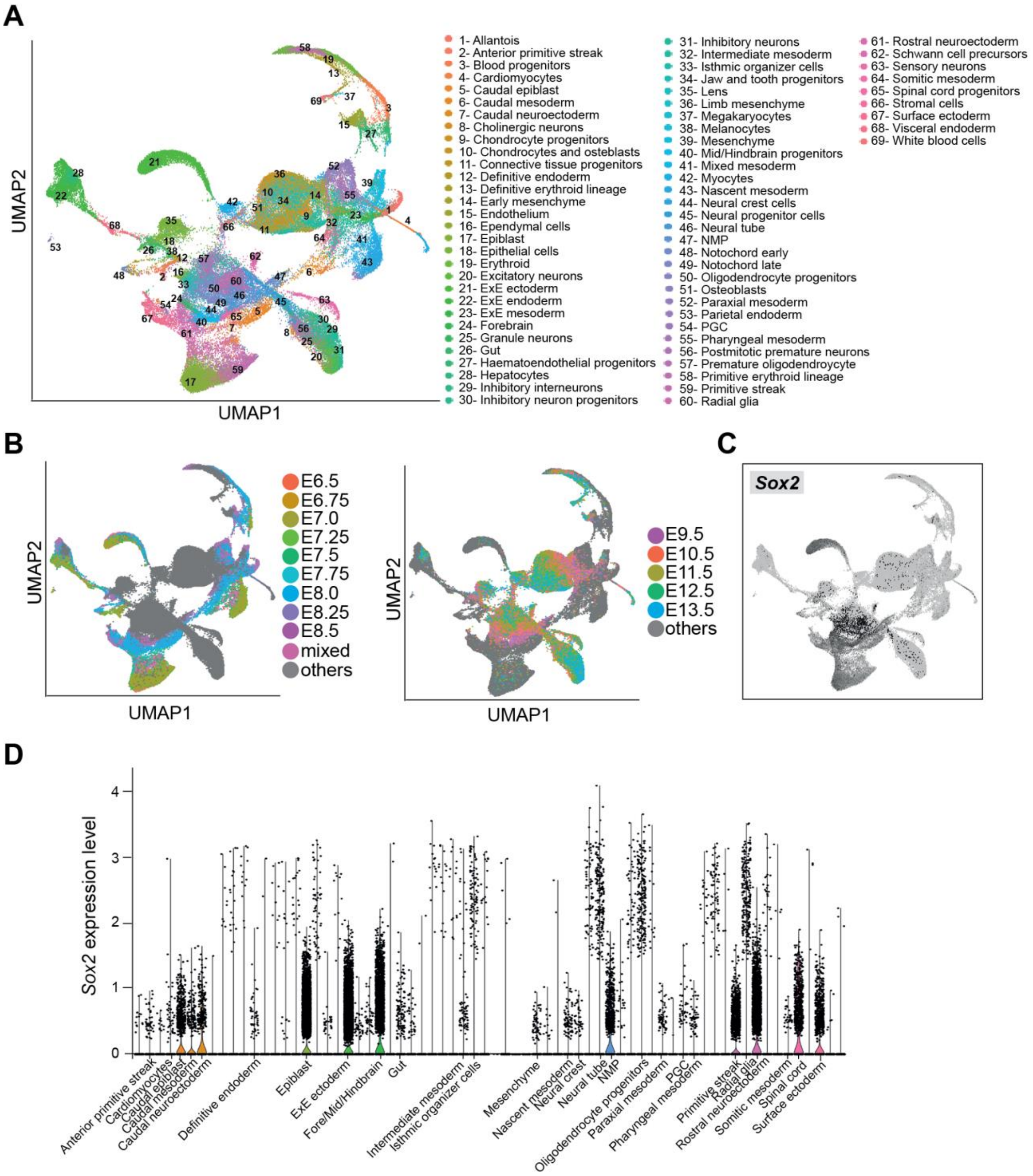

Supplementary Figure 7. Analysis of published single-cell transcriptomics atlases of murine embryos between developmental stages E6.5 and E13.5. A, Merged UMAP plot of reference single-cell transcriptome atlases of murine embryonic cells between E6.5 and E13.5[30,31]. Each atlas was randomly sampled to 50,000 total cells and dataset integration was performed using Seurat[32], keeping the cell type annotations as provided in the original atlases. **B,** UMAPs of the merged embryo atlas showing the embryonic stages for early (Pijuan-Sala et al., 2019[30], left panel) and late (Cao et al., 2019[31], right panel) datasets. **C,** UMAP shows the expression of *Sox2* in the merged embryo atlas. **D,** Individual violin plots depict *Sox2* expression across all embryonic cell types of the merged embryo

atlas. Cell types expressing moderate to high *Sox2* levels are outlined. Source data are provided as a Source Data file.

Supplementary Figure 8

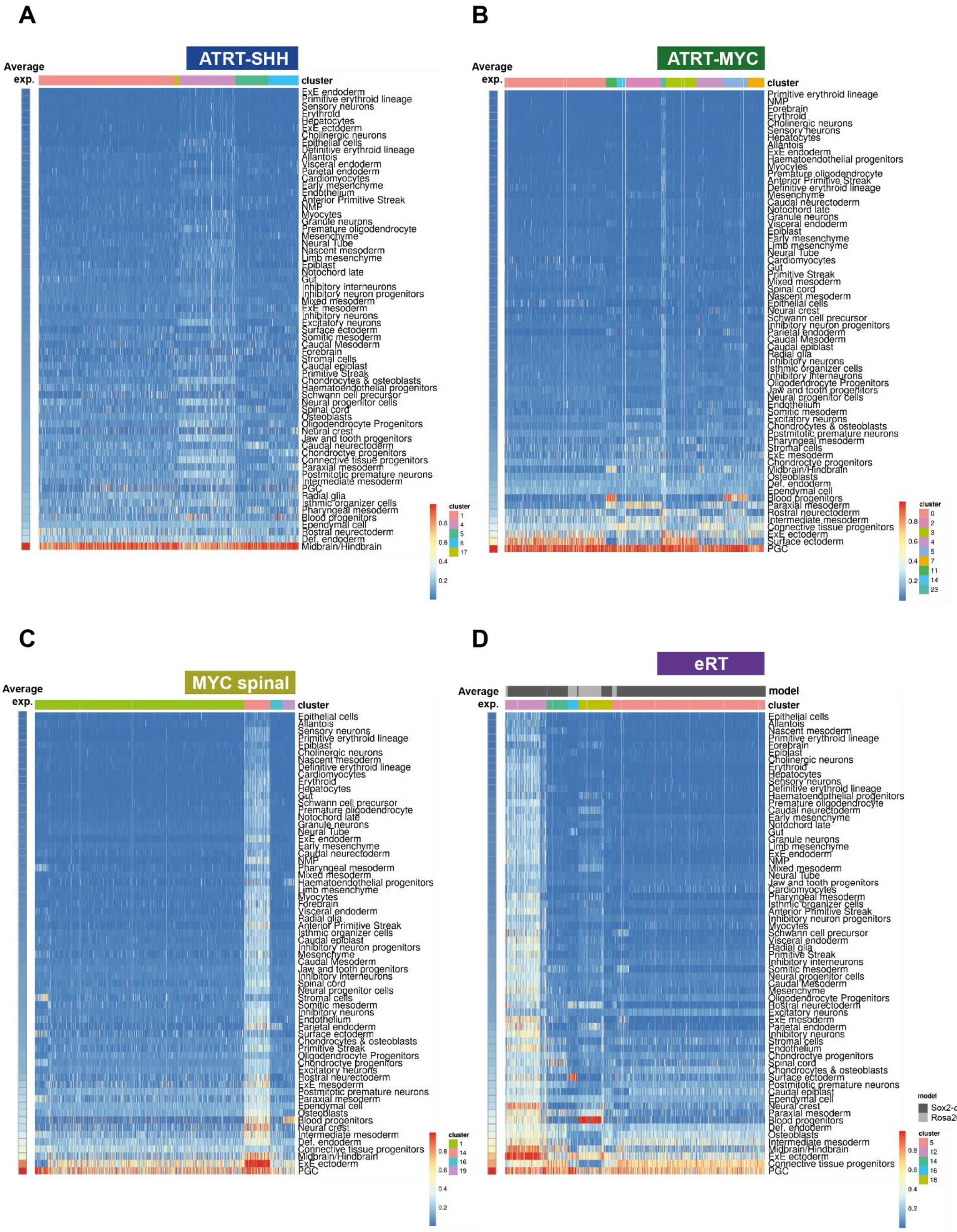

Supplementary Figure 8. A logistic regression approach identifies candidate cells of origin for RT of the MYC and SHH subgroups. Similarity scores calculated by logistic regression are shown for murine RT tumor cells of ATRT-SHH (**A**) and MYC tumors of each subtype, ATRT-MYC (**B**), MYC spinal (**C**) and eRT (**D**). Rows represent annotated embryonal cell types of the reference embryo atlas; columns show the tumor cell clusters of our single-cell murine data. Colors represent the probability for

high (red) to low similarity (blue). **D,** shows on top the annotation to the two mouse models, Rosa26-*cre* or Sox2-*cre*. For **A, B,** and **C,** all cells are derived from Rosa26-*cre* mice. Source data are provided as a Source Data file.

Supplementary Figure 9

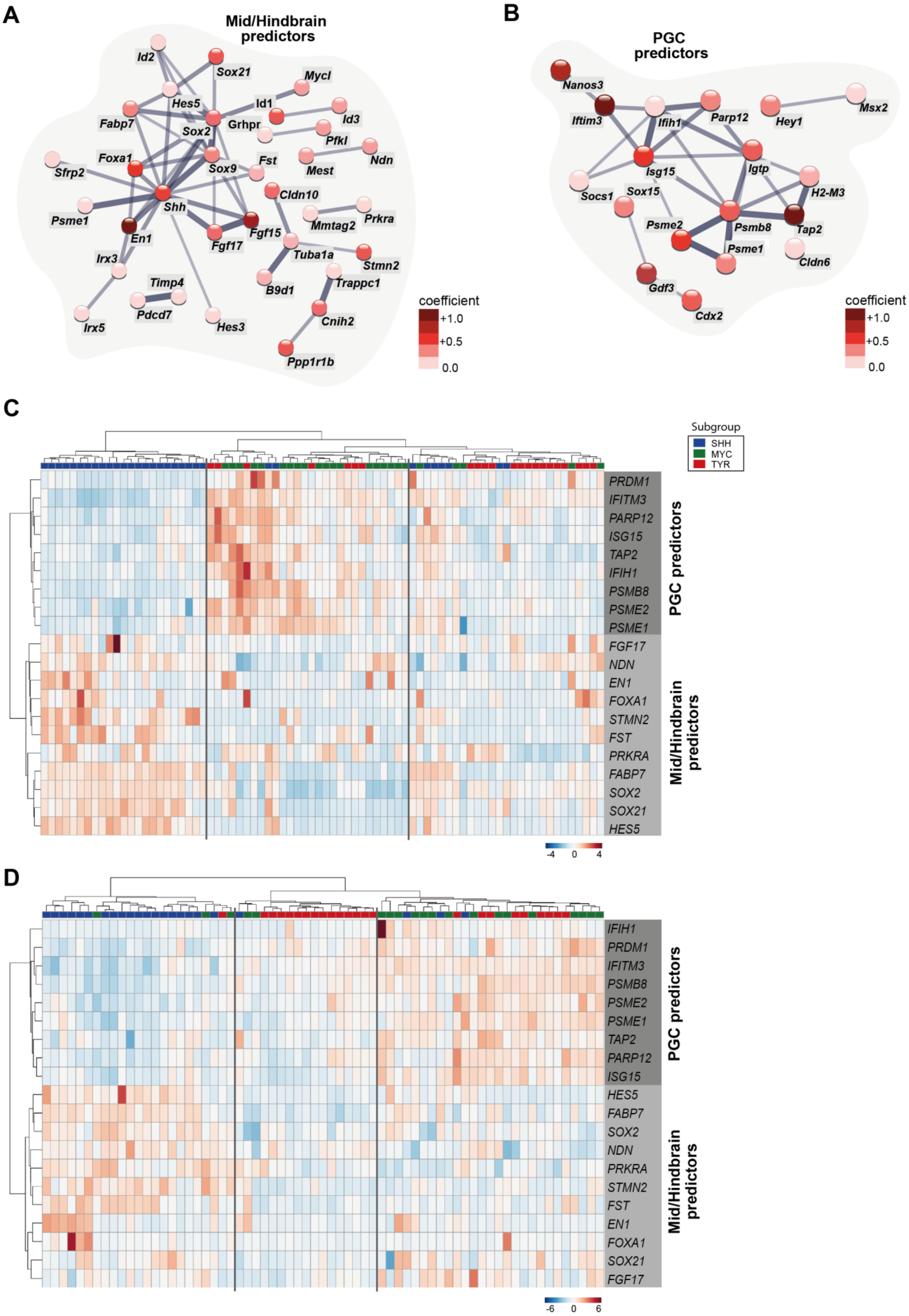

Supplementary Figure 9. Functional gene network analysis reveals a tight connection between predictors genes found by the logistic regression. A, STRING gene network of predictor genes that build the logistic functions. Colors represent high (dark red) to low (light red) coefficient scores. **B,** STRING gene network of PGC predictor genes that build the logistic functions. Colors represent high (dark red) to low (light red) coefficient scores. **C,** Unsupervised hierarchical clustering of 85 human ATRT samples[34] segregated according to the expression of selected PGC and mid/hindbrain progenitor genes. Four samples (T17, T32, T16 and T18) were excluded from the heatmap since they showed an outlier gene expression profile. **D,** Unsupervised hierarchical clustering of 67 human ATRT samples[7,85] segregated according to the expression of selected PGC and mid/hindbrain progenitor genes. For both clusterings, euclidean distance and Ward.D2 methods were used. Molecular subgroups (SHH/MYC/TYR) were taken from the original publications. Source data are provided as a Source Data file.

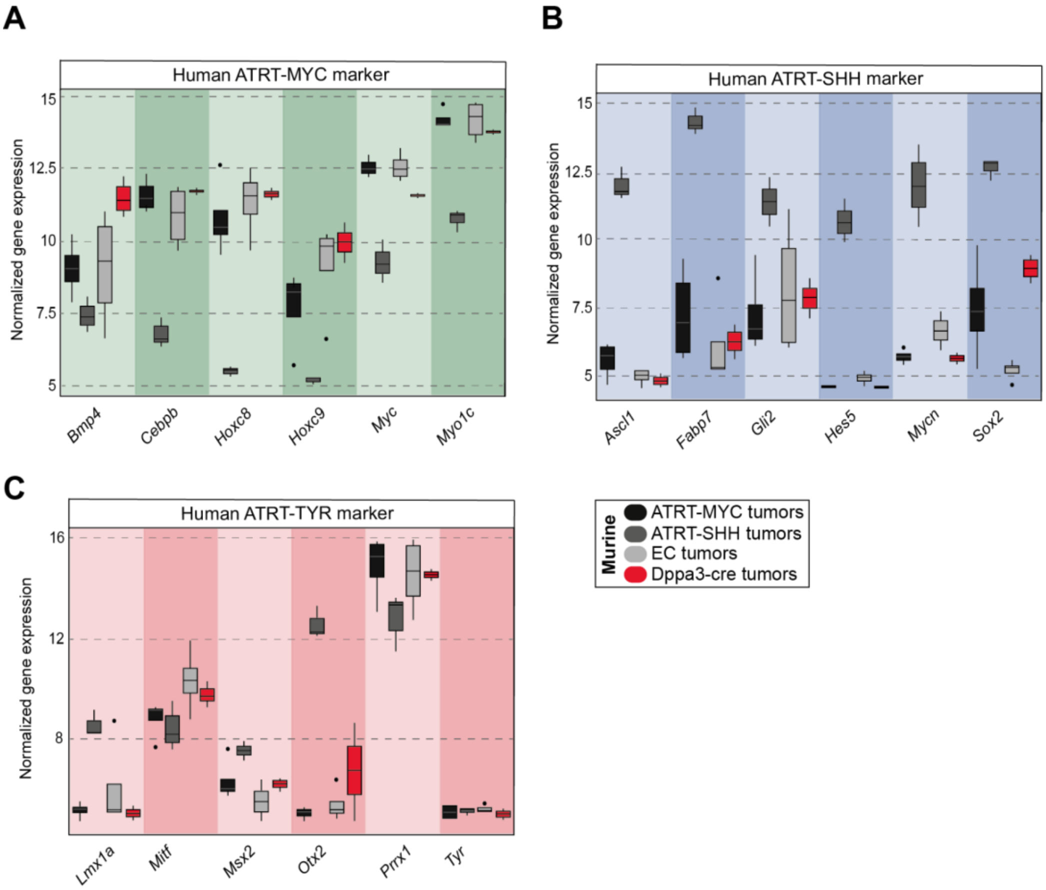

Supplementary Figure 10. RT generated in *Dppa3-cre::Smarcb1*$^{Fl/+}$ mice show expression of RT subgroup markers comparable to murine tumors. Boxplots displaying the expression of known gene markers of each human ATRT subgroup (ATRT-MYC in **A**, ATRT-SHH in **B** and ATRT-TYR in **C**), in RNAseq data from two murine tumors derived from *Dppa3-cre:: Smarcb1*$^{Fl/+}$ mice and murine RT tumors from GSE137633 (3 ATRT-SHH, 4 ATRT-MYC and 4 EC tumors). For each box, the lower and upper bounds represent the 25th and 75th percentiles; the center corresponds to the 50th percentile (median). The upper whisker extends to the largest value no further than 1.5 * IQR from the bound (where IQR is the interquartile range, or distance between the 25th and 75th percentiles). The lower whisker extends to the smallest value at most 1.5 * IQR of the bound. Data beyond the end of the whiskers are called outlying points and are plotted individually. Source data are provided as a Source Data file.

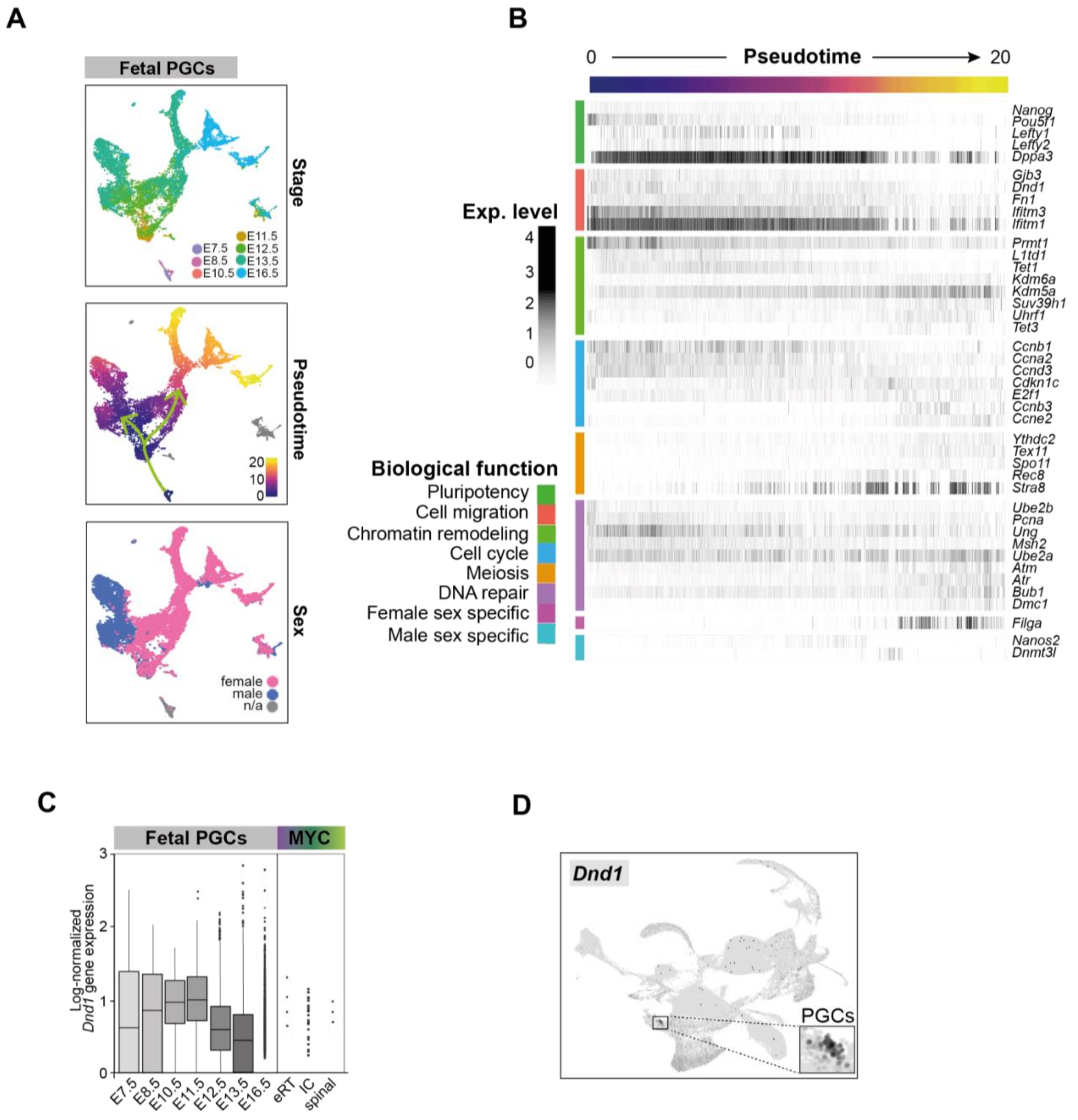

Supplementary Figure 11. Analysis of single-cell transcriptomic datasets from PGCs ranging from E7.5 to E16.5. A, Trajectory analysis and pseudotime ordering of merged PGCs [41,42] using Monocle[31] links developmental stages (up), cellular differentiation (middle) and indicates branch splitting (bottom UMAP). **B,** Heatmap of normalized expression levels of PGC-specific genes separated into different biological categories in pseudotime-ordered PGCs. **C,** Box plots depict the log-normalized expression of *Dnd1* across the indicated developmental stages of fetal PGCs and tumor cells of the three MYC subtypes. For each category, the following cell numbers are examined: E7.5=244, E8.5=109, E10.5=70, E11.5=953, E12.5=4365, E13.5=6593, E16.5=2769, eRT=1050, IC=10996,

spinal=1904. For each box, the lower and upper bounds represent the 25th and 75th percentiles; the center corresponds to the 50th percentile (median). The upper whisker extends to the largest value no further than 1.5 * IQR from the bound (where IQR is the interquartile range, or distance between the 25th and 75th percentiles). The lower whisker extends to the smallest value at most 1.5 * IQR of the bound. Data beyond the end of the whiskers are called outlying points and are plotted individually. **D,** UMAP represents the expression of *Dnd1* in the merged embryo atlas. Boxed area is magnified and depicts cells annotated as PGCs. Source data are provided as a Source Data file.

Supplementary Figure 12

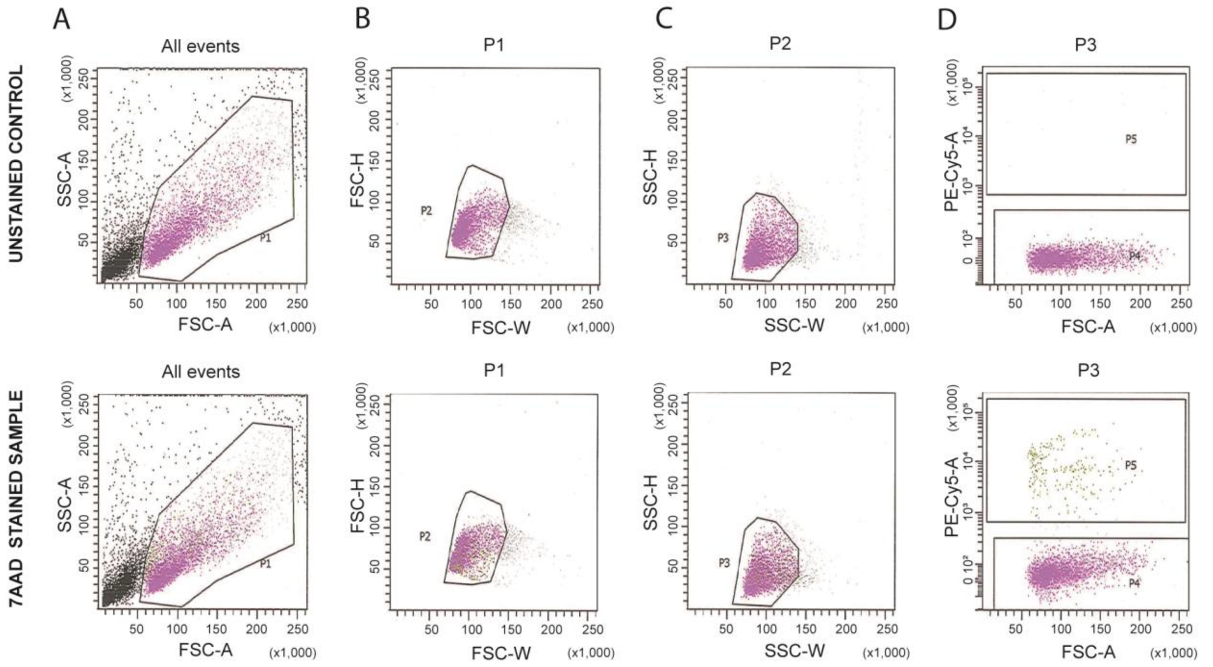

Supplementary Figure 12. FACS gating strategy used to isolate living single cells from tumor samples. A, Panels showing all events detected in the single-cell suspension unstained (upper) and after 7AAD staining. P1 gating excludes cell debris from the samples. **B and C,** gating for excluding doublets**. D,** Panel showing gating that allows the separation of living (P4) from dead (P5) cells. In the lower panel, P5 cells are 7AAD+, and will be excluded from the analysis.

Supplementary Tables

Supplementary Table 1, related to **Fig. 1** and **Supplementary Fig. 1-3**. Sample summary of all experimental mouse models. IC, intracranial; EC, extracranial; Sp, spinal; w; weeks; d; days; Ex.x; embryonic day x.x; P, postnatal n; number; *, Moreno et al, 2014.

	Genotype	*cre* expression/induction at:	n [Mice]	Phenotype	n [Tumors]	Penetrance	Observation time, Tumor onset
Constitutive	*Nestin-cre::Smarcb1*$^{fl/fl}$	E11.5	lethal	Brain hyperproliferative regions at E14.5	-	-	E14.5
	hGFAP-cre::Smarcb1$^{fl/fl}$ *****	E13.5	viable [d]	cerebellar hypoplasia	-	-	E14.5
	Math1-cre::Smarcb1$^{fl/fl}$	E10.5	53	severe motor defects & ataxia after P14, cerebellar hypoplasia	-	-	average 34 w
	Olig1-cre::Smarcb1$^{fl/fl}$	E12.5	lethal	Brain hyperproliferative regions at E14.5	-	-	E14.5
	Sox2-cre::Smarcb1$^{fl/fl}$	E6.5	lethal	abortion at early pregnancy	-	-	-
	Nestin-cre::Smarcb1$^{fl/+}$	E11.5	60	no phenotype	-	-	average 70 w
	hGFAP-cre::Smarcb1$^{fl/+}$	E13.5	56	different tumor entities	6	11.00%	maximal 105 w, average 72 w
	Math1-cre::Smarcb1$^{fl/+}$	E10.5	75	different tumor entities	3	4.00%	maximal 72, average 69 w
	Olig1-cre::Smarcb1$^{fl/+}$	E12.5	60	no phenotype	-	-	average 70 w
	Sox2-cre::Smarcb1$^{fl/+}$	E6.5	106	**rhabdoid tumor formation**	19<br>6 IC/10 EC/3 Sp	18.00%	maximal 60 w, average 40.5 w
Inducible	*Sox2-cre*^{ERT2}*::Smarcb1*$^{fl/fl}$	E6.5	64	**rhabdoid tumor formation**	14<br>9 IC/3 EC/2 Sp	22.00%	maximal 51 w, average 27 w
	Rosa26-cre^{ERT2}*::Smarcb1*$^{fl/fl}$	E6.5	115	**rhabdoid tumor formation**	46<br>30 IC/11 EC/5 Sp	40.00%	maximal 54 w, average 17 w

Supplementary Table 2, related to **Fig. 2** and **Supplementary Figure 4**. Sample summary of GEMM samples processed through gene expression profiling.

cre-driver	mouse model	ID	IC (intracranial) or EC (extracranial)	body location	subgroup	sex	Age (weeks)
Rosa-Cre	inducible	867	EC	gland thorax	MYC	M	23
Rosa-Cre	inducible	874	EC	head	MYC	M	17
Rosa-Cre	inducible	871	EC	gland	MYC	F	51
Rosa-Cre	inducible	748	IC	brain SVZ	SHH	F	15
Rosa-Cre	inducible	863	IC	trigeminal	MYC	F	22
Rosa-Cre	inducible	1070	IC	brain,lateral	MYC	M	11
Rosa-Cre	inducible	961	IC	brain SVZ	SHH	F	6
Rosa-Cre	inducible	867	IC	brain, basal	SHH	M	23
Rosa-Cre	inducible	822	IC	trigeminal	MYC	F	23
Rosa-Cre	inducible	991	IC	brain, lateral	MYC	M	14
Rosa-Cre	inducible	1112	IC	spinal	MYC	M	10
Rosa-Cre	inducible	64	EC	mouth	MYC	M	13
Rosa-Cre	inducible	63	EC	facial,maxilar	MYC	F	13
Rosa-Cre	inducible	63	IC	brain, basal	SHH	F	13
Rosa-Cre	inducible	74	IC	brain SVZ	SHH	M	20
Rosa-Cre	inducible	128	IC	brain SVZ	SHH	M	11
Rosa-Cre	inducible	355	IC	brain SVZ	SHH	F	16
Rosa-Cre	inducible	407	IC	eye	MYC	M	11
Rosa-Cre	inducible	401	IC	brain SVZ	SHH	M	13
Rosa-Cre	inducible	444	IC	brain lateral	MYC	F	16
Rosa-Cre	inducible	446	IC	brain lateral	MYC	M	20
Rosa-Cre	inducible	407	EC	lip	MYC	M	11
Rosa-Cre	inducible	445	IC	spinal	MYC	M	18
Rosa-Cre	inducible	147	IC	brain	MYC	F	9
Sox2-Cre	inducible	396	EC	mouth	MYC	M	13
Sox2-Cre	inducible	391	EC	shoulder	MYC	F	29
Sox2-Cre	inducible	183	IC	brain, basal	SHH	M	48
Sox2-Cre	inducible	394	IC	brain, basal	MYC	F	22
Sox2-Cre	inducible	391	IC	brain, lateral	MYC	F	29
Sox2-Cre	inducible	456	IC	brain, basal	SHH	F	29
Sox2-Cre	inducible	235	IC	brain, basal	MYC	M	17
Sox2-Cre	inducible	78	IC	eye	MYC	M	20
Sox2-Cre	inducible	97	IC	brain, basal	MYC	M	34
Sox2-Cre	constitutive	125	EC	mouth	MYC	F	22
Sox2-Cre	constitutive	171	EC	mouth	MYC	F	50
Sox2-Cre	constitutive	99	EC	subcutaneous, fat tissue	MYC	M	42
Sox2-Cre	constitutive	288	EC	facial,maxilar	MYC	F	45
Sox2-Cre	constitutive	264	EC	lip	MYC	F	49
Sox2-Cre	constitutive	203	EC	gland thorax	MYC	M	22
Sox2-Cre	constitutive	137	IC	brain, basal	MYC	F	51
Sox2-Cre	constitutive	247	IC	spinal	MYC	M	43

Supplementary Table 3, related to **Fig. 3** and subsequent **Figures**. Sample summary of GEMM samples processed through single-cell RNA sequencing. F, female; M, male; IC, intracranial; EC, extracranial; SVZ, subventricular zone; No., number.

Mouse model	Mouse ID	Sex	Age [weeks]	Tumor location	Subgroup	Estimated No. of cells	Saturation in %	No. of reads	Mean reads / cell	Median genes / cell	No. of genes (after quality control)	No. of cells (after quality control)
Rosa26-cre^{ERT2}::Smarcb1$^{fl/fl}$	148	F	12	IC; basal	ATRT-MYC	361	96.1	231 Mio	642015	2016	13797	348
Rosa26-cre^{ERT2}::Smarcb1$^{fl/fl}$	1132	F	19	IC; lateral of cerebellum	ATRT-MYC	222	93.2	77 Mio	348346	2208	12376	194
Rosa26-cre^{ERT2}::Smarcb1$^{fl/fl}$	1132	F	19	IC; trigeminal nerve	ATRT-MYC	690	76.6	47 Mio	68906	981	12889	455
Rosa26-cre^{ERT2}::Smarcb1$^{fl/fl}$	1145	M	21	IC; trigeminal nerve	ATRT-MYC	2271	70.9	60 Mio	26638	802	13371	2267
Rosa26-cre^{ERT2}::Smarcb1$^{fl/fl}$	1225	M	27	IC; basal	ATRT-MYC	15282	46.8	513 Mio	33583	2000	18889	15278
Rosa26-cre^{ERT2}::Smarcb1$^{fl/fl}$	1192	M	14	IC; SVZ/striatal	ATRT-SHH	3864	57.9	80 Mio	20725	963	15096	3859
Rosa26-cre^{ERT2}::Smarcb1$^{fl/fl}$	1237	M	16	IC; SVZ/striatal	ATRT-SHH	1804	74.00	74 Mio	41536	1352	14483	1798
Rosa26-cre^{ERT2}::Smarcb1$^{fl/fl}$	1148	M	16	EC; gland	eRT	2153	77.90	134 Mio	62635	1808	13670	2143
Sox2-cre^{ERT2}::Smarcb1$^{fl/fl}$	568	M	29	EC; jaw	eRT	2056	87.10	85 Mio	41365	624	12949	2042
Sox2-cre^{ERT2}::Smarcb1$^{fl/fl}$	593	F	22	EC; shoulder	eRT	4020	63.40	55 Mio	13899	942	13738	4020
Rosa26-cre^{ERT2}::Smarcb1$^{fl/fl}$	1172	F	19	IC; spinal	MYC spinal	1492	88.30	62 Mio	41972	977	13268	1469
Rosa26-cre^{ERT2}::Smarcb1$^{fl/fl}$	1177	M	26	IC; spinal	MYC spinal	6236	89.70	141 Mio	22684	411	12746	5744
Rosa26-cre^{ERT2}::Smarcb1$^{fl/fl}$	1232	F	17	IC; spinal	MYC spinal	6744	66.60	163 Mio	24290	1277	15277	6740

Supplementary Table 4. Cell numbers per molecular subgroup, per cluster (single-cell RNAseq murine datasets).

**Cluster**	**eRT**	**ATRT-MYC**	**MYC-spinal**	**ATRT-SHH**
**0**	1497	4280	2090	1001
**1**	1299	2662	1533	982
**2**	976	1517	1465	821
**3**	705	1301	1410	796
**4**	680	1133	1330	411
**5**	619	1029	1120	246
**6**	421	923	1088	242
**7**	386	703	1016	238
**8**	327	601	939	226
**9**	306	586	340	134
**10**	264	569	306	115
**11**	171	464	305	91
**12**	167	438	258	90
**13**	166	389	200	89
**14**	85	371	187	83
**15**	56	319	186	66
**16**	43	299	96	51
**17**	37	252	42	52
**18**		229	42	
**19**		179		
**20**		166		
**21**		53		
**22**		52		

Supplementary Table 5. Permutation test p-values, for all subgroups.

SHH		MYC_IC		eRT		MYC_Spinal	
**celltype**	**p_val**	**celltype**	**p_val**	**celltype**	**p_val**	**celltype**	**p_val**
Midbrain/Hindbrain	0,021	PGC	0,024	PGC	0,024	PGC	0,024
Def. endoderm	0,027	Surface ectoderm	0,034	Connective tissue progenitors	0,028	ExE ectoderm	0,03
Rostral neurectoderm	0,032	ExE ectoderm	0,051	ExE ectoderm	0,05	Midbrain/Hindbrain	0,039
Ependymal cell	0,053	Connective tissue progenitors	0,054	Midbrain/Hindbrain	0,051	Connective tissue progenitors	0,051
Blood progenitors	0,078	Intermediate mesoderm	0,083	Intermediate mesoderm	0,088	Def. endoderm	0,085
Pharyngeal mesoderm	0,083	Rostral neurectoderm	0,108	Osteoblasts	0,096	Intermediate mesoderm	0,095
Isthmic organizer cells	0,111	Paraxial mesoderm	0,111	Def. endoderm	0,121	Blood progenitors	0,123
PGC	0,136	Ependymal cell	0,12	Blood progenitors	0,129	Neural crest	0,123
Radial glia	0,14	Blood progenitors	0,121	Paraxial mesoderm	0,134	Osteoblasts	0,138
Intermediate mesoderm	0,173	Def. endoderm	0,183	Neural crest	0,167	Ependymal cell	0,172
Paraxial mesoderm	0,183	Midbrain/Hindbrain	0,189	Ependymal cell	0,18	Paraxial mesoderm	0,18
Postmitotic premature neurons	0,199	Osteoblasts	0,189	Caudal epiblast	0,183	Postmitotic premature neurons	0,189
Jaw and tooth progenitors	0,218	Chondroctye progenitors	0,198	Postmitotic premature neurons	0,193	ExE mesoderm	0,197
Caudal neurectoderm	0,224	Stromal cells	0,218	Surface ectoderm	0,219	Rostral neurectoderm	0,218
Connective tissue progenitors	0,224	ExE mesoderm	0,223	Chondrocytes & osteoblasts	0,232	Excitatory neurons	0,225
Chondroctye progenitors	0,228	Pharyngeal mesoderm	0,242	Spinal cord	0,249	Chondroctye progenitors	0,232
Osteoblasts	0,27	Postmitotic premature neurons	0,271	Endothelium	0,271	Oligodendrocyte Progenitors	0,268
Neural crest	0,272	Chondrocytes & osteoblasts	0,281	Chondroctye progenitors	0,273	Primitive Streak	0,272
Oligodendrocyte Progenitors	0,282	Somitic mesoderm	0,29	Stromal cells	0,279	Chondrocytes & osteoblasts	0,277
Spinal cord	0,299	Excitatory neurons	0,295	Inhibitory neurons	0,306	Surface ectoderm	0,302
Neural progenitor cells	0,322	Endothelium	0,31	Parietal endoderm	0,316	Parietal endoderm	0,326
Schwann cell precursor	0,335	Jaw and tooth progenitors	0,353	Excitatory neurons	0,346	Endothelium	0,341
Haematoendothelial progenitors	0,361	Neural progenitor cells	0,36	ExE mesoderm	0,354	Inhibitory neurons	0,35
Chondrocytes & osteoblasts	0,373	Oligodendrocyte Progenitors	0,362	Rostral neurectoderm	0,377	Somitic mesoderm	0,368
Primitive Streak	0,386	Inhibitory interneurons	0,398	Oligodendrocyte Progenitors	0,392	Stromal cells	0,393
Caudal epiblast	0,403	Inhibitory neurons	0,417	Mesenchyme	0,42	Spinal cord	0,423
Forebrain	0,41	Isthmic organizer cells	0,417	Caudal Mesoderm	0,426	Neural progenitor cells	0,423
Stromal cells	0,41	Radial glia	0,431	Neural progenitor cells	0,438	Inhibitory interneurons	0,437
Somitic mesoderm	0,442	Caudal epiblast	0,436	Somitic mesoderm	0,447	Jaw and tooth progenitors	0,44
Caudal Mesoderm	0,463	Caudal Mesoderm	0,462	Inhibitory interneurons	0,472	Caudal Mesoderm	0,461
Surface ectoderm	0,468	Parietal endoderm	0,49	Primitive Streak	0,499	Mesenchyme	0,499
Excitatory neurons	0,512	Inhibitory neuron progenitors	0,511	Radial glia	0,509	Inhibitory neuron progenitors	0,516
ExE mesoderm	0,538	Neural crest	0,536	Visceral endoderm	0,527	Isthmic organizer cells	0,524

Inhibitory neurons	0,543	Schwann cell precursor	0,542	Schwann cell precursor	0,538	Caudal epiblast	0,529
Mixed mesoderm	0,554	Epithelial cells	0,551	Myocytes	0,549	Radial glia	0,553
Inhibitory neuron progenitors	0,559	Nascent mesoderm	0,57	Inhibitory neuron progenitors	0,555	Anterior Primitive Streak	0,553
Inhibitory interneurons	0,572	Spinal cord	0,575	Anterior Primitive Streak	0,579	Visceral endoderm	0,574
Gut	0,586	Mixed mesoderm	0,579	Isthmic organizer cells	0,586	Forebrain	0,588
Epiblast	0,595	Primitive Streak	0,615	Cardiomyocytes	0,631	Myocytes	0,625
Notochord late	0,603	Gut	0,629	Pharyngeal mesoderm	0,632	Limb mesenchyme	0,629
Neural Tube	0,64	Cardiomyocytes	0,66	Jaw and tooth progenitors	0,647	Haematoendothelial progenitors	0,643
Limb mesenchyme	0,648	Neural Tube	0,668	Mixed mesoderm	0,662	Pharyngeal mesoderm	0,656
Nascent mesoderm	0,666	Limb mesenchyme	0,67	Neural Tube	0,663	Mixed mesoderm	0,658
Mesenchyme	0,7	Early mesenchyme	0,682	NMP	0,693	NMP	0,695
Granule neurons	0,717	Epiblast	0,709	ExE endoderm	0,721	Caudal neurectoderm	0,707
Premature oligodendrocyte	0,737	Granule neurons	0,727	Granule neurons	0,739	Early mesenchyme	0,73
Myocytes	0,749	Visceral endoderm	0,739	Limb mesenchyme	0,741	ExE endoderm	0,733
NMP	0,763	Notochord late	0,747	Early mesenchyme	0,758	Notochord late	0,757
Anterior Primitive Streak	0,767	Caudal neurectoderm	0,765	Gut	0,763	Granule neurons	0,767
Endothelium	0,79	Mesenchyme	0,772	Notochord late	0,766	Neural Tube	0,768
Early mesenchyme	0,811	Definitive erythroid lineage	0,801	Premature oligodendrocyte	0,8	Schwann cell precursor	0,8
Cardiomyocytes	0,814	Anterior Primitive Streak	0,815	Caudal neurectoderm	0,822	Premature oligodendrocyte	0,815
Parietal endoderm	0,828	Premature oligodendrocyte	0,825	Haematoendothelial progenitors	0,828	Gut	0,822
Allantois	0,838	Haematoendothelial progenitors	0,827	Sensory neurons	0,837	Erythroid	0,838
Visceral endoderm	0,85	Myocytes	0,861	Definitive erythroid lineage	0,841	Hepatocytes	0,842
Definitive erythroid lineage	0,89	ExE endoderm	0,876	Hepatocytes	0,886	Cardiomyocytes	0,882
Epithelial cells	0,893	Allantois	0,886	Erythroid	0,893	Definitive erythroid lineage	0,891
Cholinergic neurons	0,905	Hepatocytes	0,91	Cholinergic neurons	0,909	Nascent mesoderm	0,912
Hepatocytes	0,928	Sensory neurons	0,925	Epiblast	0,936	Cholinergic neurons	0,933
ExE ectoderm	0,928	Erythroid	0,946	Forebrain	0,939	Epiblast	0,935
Erythroid	0,954	Cholinergic neurons	0,951	Primitive erythroid lineage	0,958	Primitive erythroid lineage	0,948
Sensory neurons	0,967	Forebrain	0,967	Nascent mesoderm	0,965	Sensory neurons	0,965
Primitive erythroid lineage	0,986	NMP	0,98	Allantois	0,978	Allantois	0,978
ExE endoderm	1	Primitive erythroid lineage	1	Epithelial cells	1	Epithelial cells	1
