# Supplementary material for: Single-cell transcriptomics identifies potential cells of origin of MYC rhabdoid tumors
Source: Nat Commun. 2022 Mar 22;13:1544. doi: 10.1038/s41467-022-29152-4 (PMC8941154; doi:10.1038/s41467-022-29152-4)
Supplement: Supplementary file 2 — Reporting Summary [file 41467_2022_29152_MOESM2_ESM.pdf]

nature portfolio

Corresponding author(s): Kornelius Kerl

Last updated by author(s): Feb 11, 2022

Reporting Summary

Nature Portfolio wishes to improve the reproducibility of the work that we publish. This form provides structure for consistency and transparency in reporting. For further information on Nature Portfolio policies, see our Editorial Policies and the Editorial Policy Checklist.

Statistics

For all statistical analyses, confirm that the following items are present in the figure legend, table legend, main text, or Methods section.

n/a	Confirmed	
☐	☒	The exact sample size (*n*) for each experimental group/condition, given as a discrete number and unit of measurement
☐	☒	A statement on whether measurements were taken from distinct samples or whether the same sample was measured repeatedly
☐	☒	The statistical test(s) used AND whether they are one- or two-sided<br>*Only common tests should be described solely by name; describe more complex techniques in the Methods section.*
☒	☐	A description of all covariates tested
☐	☒	A description of any assumptions or corrections, such as tests of normality and adjustment for multiple comparisons
☐	☒	A full description of the statistical parameters including central tendency (e.g. means) or other basic estimates (e.g. regression coefficient) AND variation (e.g. standard deviation) or associated estimates of uncertainty (e.g. confidence intervals)
☐	☒	For null hypothesis testing, the test statistic (e.g. *F*, *t*, *r*) with confidence intervals, effect sizes, degrees of freedom and *P* value noted<br>*Give P values as exact values whenever suitable.*
☒	☐	For Bayesian analysis, information on the choice of priors and Markov chain Monte Carlo settings
☒	☐	For hierarchical and complex designs, identification of the appropriate level for tests and full reporting of outcomes
☒	☐	Estimates of effect sizes (e.g. Cohen's *d*, Pearson's *r*), indicating how they were calculated

Our web collection on statistics for biologists contains articles on many of the points above.

Software and code

Policy information about availability of computer code

Data collection

Flow Cytometry (FACS Canto II flow cytometry 280 system)
Ventana BenchMark XT (Roche Diagnostics)
Vectra® 3.0 imaging system (AKOYA Biosciences)
Bioanalyzer (Software Agilent 2100; Agilent Technologies, Inc)
Affymetrix GeneChip™ Mouse Gene 2.0 ST Array
Fluorescence-activated cell sorting (BD FACS Aria II)
Tapestation 2000 (Agilent Technologies, Inc)
Next-Seq 500 sequencing platform
Affymetrix U133 plus 2.0 human Array
Next-Seq 2000 sequencing System

Data analysis

Multiscan Ascent microplate reader (Thermo Electron Corporation).
FlowJo 10.1r1 (FlowJo LLC)
Graph Pad Prism 7.0
Phenochart™ version 1.1.0 and InForm® version 2.1
10X Genomics software Cell Ranger (version 2.0.2)
Murine and human tumors gene expression data: R (version 3.4.2) and Bioconductor (version 3.6) using packages such as sva(v3.30.1), biomaRt(v2.38.0), limma(v3.38.3).
Analysis of scRNA-seq data was performed in R (version 3.6.1), using the packages Seurat (v3.1.3), glmnet(v3.0.2), Monocle 3(v0.2.0).
For bulk RNA-seq of BT16 cell line: FastQC(v0.11.8), MultiQC(v1.7), Salmon(v0.14.1). Further analyses were performed in R(v3.4.2) and Bioconductor (v3.6) with packages tximport(v1.10.1), DESeq2(v1.22.2).
For microarray data of human RTs: R(v3.4.2) and Bioconductor (v3.6) with packages beadarray(v2.32.0), IlluminaV4db(v1.26.0), sva(v3.30.1).
DNA methylation analysis: adapted from methyLiftover(https://github.com/Christensen-Lab-Dartmouth/methyLiftover)

Dppa3-cre::Smarcb1Fl/+ mice: STAR (version 2.7.9a).

Code used to analyze the datasets is publicly available at https://github.com/martaint/AGKerl_RTcoo_analysis_scripts.

For manuscripts utilizing custom algorithms or software that are central to the research but not yet described in published literature, software must be made available to editors and reviewers. We strongly encourage code deposition in a community repository (e.g. GitHub). See the Nature Portfolio guidelines for submitting code & software for further information.

Data

Policy information about availability of data

All manuscripts must include a data availability statement. This statement should provide the following information, where applicable:

- Accession codes, unique identifiers, or web links for publicly available datasets
- A description of any restrictions on data availability
- For clinical datasets or third party data, please ensure that the statement adheres to our policy

Single-cell RNA-seq data generated in this study have been deposited in NCBI's Gene Expression Omnibus and are accessible through GEO Series accession numbers GSE141532 (for ATRT-SHH and ATRT-MYC samples) and GSE188815 (for eRT and MYC spinal samples). Gene expression array data of the murine models generated in this study are available through GEO Series accession number GSE188654. Two human ATRT datasets of gene expression data (Birks et al., 201185; Johann et al., 20167) were retrieved in GEO under accession numbers GSE70678 and GSE28026. Early stages mouse embryo data were retrieved from https://github.com/MarioniLab/EmbryoTimecourse2018 and https://oncoscape.v3.sttrcancer.org/atlas.gs.washington.edu.mouse.rna/downloads. Mouse embryonic germ cells data from Mayère et al. is available in GEO under accession number GSE136220. Bulk RNA-sequencing data of human rhabdoid tumor cell lines is deposited under GSE71505. Illumina array data of human ATRT samples from Torchia et al. can be found in EGA at EGAD00010000789, EGAD00010000790 and EGAD00010001546. Whole-genome bisulfite sequencing (WGBS) data for human PGCs was retrieved from GEO, with accession number GSE63818. For human RTs, we used methylation profiles (Illumina HumanMethylation450 BeadChip (450k) array) from the published dataset accessible in GEO:GSE70460. Bulk RNA-seq data of Dppa3-cre::Smarcb1Fl/+ mice generated in this study have been deposited in GEO with accession number GSE188816. A published dataset of bulk RNA-seq of murine ATRT tumors was retrieved from GEO (GSE137633).

Field-specific reporting

Please select the one below that is the best fit for your research. If you are not sure, read the appropriate sections before making your selection.

☒ Life sciences ☐ Behavioural & social sciences ☐ Ecological, evolutionary & environmental sciences

For a reference copy of the document with all sections, see nature.com/documents/nr-reporting-summary-flat.pdf

Life sciences study design

All studies must disclose on these points even when the disclosure is negative.

Sample size	No statistical methods were used to predetermine sample sizes. Sample sizes were determined to be adequate based on statistical testing of magnitude and consistency between groups in the experimental phase and were in line with previous publications.
Data exclusions	No samples were excluded from the study. For scRNA-seq of murine tumors, we filtered out cells having a number of genes < 200 and percent.mito > 25%. For mouse embryos, only high-quality cells determined by the original studies were considered. In order to reduce computational time, we randomly sampled 50,000 cells from each of the two embryo datasets, making sure that the relative proportions of the different cell types were maintained before and after subsampling. In constructing the training data for the logistic regression model, one cell type (lens) was excluded, as composed by only three cells. In the human RT tumors data from Torchia et al., four samples (T17, T32, T16 and T18) were excluded from the heatmap since they showed an outlier gene expression profile.
Replication	For single-cell transcriptome analyses of murine tumors, at least 3 biological samples were considered for any of the MAC RT tumors, and 2 biological samples for the SHH tumors (as previously published in Melcher et al., 2020).<br>For in vitro experiments at least 2 technical triplicates were considered (if not otherwise stated). Depending on the method used, several cell lines were analyzed (up to eight RT cell lines corresponding to the SHH or MYC RT subgroup).<br>For in vivo analyses, between 6 and 8 mice were considered per condition.
Randomization	For in vivo experiments, after tumor engraftment mice were randomized into two different groups (untreated control and treated mice).
Blinding	No blinding was performed in the data analyses.

Reporting for specific materials, systems and methods

We require information from authors about some types of materials, experimental systems and methods used in many studies. Here, indicate whether each material, system or method listed is relevant to your study. If you are not sure if a list item applies to your research, read the appropriate section before selecting a response.

nature portfolio | reporting summary

March 2021

Materials & experimental systems

n/a	Involved in the study
☐	☒ Antibodies
☐	☒ Eukaryotic cell lines
☒	☐ Palaeontology and archaeology
☐	☒ Animals and other organisms
☒	☐ Human research participants
☒	☐ Clinical data
☒	☐ Dual use research of concern

Methods

n/a	Involved in the study
☒	☐ ChIP-seq
☒	☐ Flow cytometry
☒	☐ MRI-based neuroimaging

Antibodies

Antibodies used

anti-KI67 (Abcam, #ab15580), 1:100, Crispr- Cas9 genome editing-based Knock out validation
anti-SMARCB1 (Clone 25/BAF47, BD Bioscience, #612110), 1:50. This antibody is routinely tested by western blot analysis. Other applications were tested at BD Biosciences Pharmingen during antibody development only or reported in the literature.
anti-Hepar (OCH1E5, Roche, #1760-4350), 1:100. Roche antibody for Ventana staining platform, In-house validation.
Inhibin alpha (MRQ-63, Roche, #760-6081). Roche antibody for Ventana Staining platform, In-house validation.
CD3 (SP7, Abcam, #ab16669), 1:50. Validated on Flow- cytometry, IHC, WB.
CD45 (Abcam, #ab10558), 1:100. Validated on Flow- cytometry, IHC, WB.
Sox2(Abcam, #ab97959), 1:200. Validated for ICC, IHC, WB.

Validation

All antibodies used are commercially available and were validated by the manufacturer (see above)

Eukaryotic cell lines

Policy information about cell lines

Cell line source(s)

BT16 cells (RRID: CVCL_M156; ATRT-MYC) were provided by Prof. Dr. Martin Hasselblatt (University Hospital Münster, Germany). These cells were not commercially aquired. G401 cells (RRID: CVCL_0270; extracranial rhabdoid tumor of the kidney) were purchased from ATCC® (#CRL-1441™). Chla02 were purchased from ATCC® (#CRL-3020). 310-FHTC and 311-FHTC cells were purchased from Fred Hutchinson. Chla266 and BT12 were a gift from Dr. Mark Remke (University Hospital Düsseldorf, Düsseldorf, who had requested them to the "Childhood Cancer Repository " (https://www.cccells.org/tables/Brain.php). A204 cells were provided by Prof. Dr. Martin Hasselblatt (University Hospital Münster, Münster). These cells were not commercially available.

Authentication

Cell lines were authenticated by STR profiling through the Institute for Forensic Medicine (University of Münster)

Mycoplasma contamination

All cell lines were tested in the beginning of any experiment and in between (every ~8weeks) using mycoplasma PCR in house, and were negative without any exception.

Commonly misidentified lines
(See ICLAC register)

The study did not involve misidentified lines.

Animals and other organisms

Policy information about studies involving animals; ARRIVE guidelines recommended for reporting animal research

Laboratory animals

Nestin-cre , hGFAP-cre , Math1-cre2, Olig1-cre, Sox2-cre, Sox2-creERT2 , Rosa26-creERT2 and Smarcb1fl/fl mice were obtained from the Jackson Laboratory (https://www.jax.org/). C57BL/6-Dppa3em1(IRES-Cre)Smoc mice were obtained from Shanghai Model organisms (#NM-KI-00040). Mice were maintained on a C57BL/6 background. By crossing the Smarcb1fl/fl strain with diverse knock-in mouse lines harboring the cre or creERT2 coding region under the control of different cell specific (Nestin, hGFAP, Math1, Olig1, Sox2, Dpp3a) or ubiquitous (Rosa26) promoters, we obtained Nestin-cre::Smarcb1fl/fl, hGFAP-cre::Smarcb1fl/fl, Math1-cre::Smarcb1fl/fl, Sox2-cre::Smarcb1fl/fl, Olig-cre::Smarcb1fl/fl, Sox2-creERT2::Smarcb1fl/fl, Dppa3-cre:: marcb1fl/fl and Rosa26-creERT2::Smarcb1fl/fl mice. Fate mapping experiments were carried out using Sox2-creERT2::Smarcb1fl/fl mice crossed with a R26-stop-EYFP reporter line. Males and females were used.
Xenograft model experiments were carried out in males and females aged 8 to 12 week old immune-compromised NOD/SCID mice (Jackson Laboratory, https://www.jax.org/). Animals were anesthetized using Isoflurane (Isoflurane Forene®, Abbvie, #B506) and transplanted into the flank with either G401 cells or cells of a freshly obtained relapsed patient sample. Tumor growth was monitored twice a week with a manual caliper. Tumor volumes were calculated using the formula: a * (b)2 / 2 (a and b considered as the two planes of a tumor). If tumors reached 3,000 mm3 or necrosis was observed, experiments were stopped before the end of the observation period.

Wild animals

The study did not involve wild animals.

Field-collected samples

The study did not involve field-collected samples.

Ethics oversight	A single dose (50 mg/kg of body weight) of Tamoxifen (Sigma-Aldrich, #T56489) was administered intraperitoneally to pregnant Sox2-creERT2::Smarcb1fl/fl and Rosa26-creERT2::Smarcb1fl/fl mice at E6.5 post-coitum (post-coital plug observation was considered as day 0.5). Mice were monitored daily for at least 30 weeks until neurologic symptoms (impaired gait, tilt head, stereotypy) or distress signs (lethargy, weakness) were observed. Mice were sacrificed by cervical dislocation under isofluran anesthesia. All animal procedures were performed according to the guidelines provided by the local regulatory authorities (reference number TVA-84-02.04.2015.A088; TVA-84-02.04.2016.A066; Government of NRW, Germany).<br>For Xenograft models, protocols and animal housing were in accordance with all guidelines provided by the local regulatory authorities (reference number TVA-84-02.04.2012.A241 and TVA-84-02.04.2014.A279; Government of NRW, Germany).

Note that full information on the approval of the study protocol must also be provided in the manuscript.
